# Differential seedling growth and tolerance indices reflect drought tolerance in cotton

**DOI:** 10.1186/s12870-022-03724-4

**Published:** 2022-07-11

**Authors:** Tahir Mahmood, Muhammad Shahid Iqbal, Hongge Li, Mian Faisal Nazir, Shiguftah Khalid, Zareen Sarfraz, Daowu Hu, Chen Baojun, Xiaoli Geng, Sani Muhammad Tajo, Washu Dev, Zubair Iqbal, Pan Zhao, Guanjing Hu, Xiongming Du

**Affiliations:** 1 State Key Laboratory of Cotton Biology, Institute of Cotton Research (ICR), Chinese Academy of Agricultural Sciences (CAAS), Anyang, 455000 China; 2 Shenzhen Branch, Guangdong Laboratory of Lingnan Modern Agriculture, Genome Analysis Laboratory of the M inistry of Agriculture and Rural Affairs, Agricultural Genomics Institute at Shenzhen, Chinese Academy of Agricultural Sciences, Shenzhen, 518120 China; 3 School of Agricultural Sciences, Zhengzhou University, Zhengzhou, 450001 China

**Keywords:** *Gossypium* spp, Cotton, Drought tolerance, Shoot growth, Root growth, Seedling growth rate, Drought stress response indices

## Abstract

**Background:**

Cotton production is adversely effected by drought stress. It is exposed to drought stress at various critical growth stages grown under a water scarcity environment. Roots are the sensors of plants; they detect osmotic stress under drought stress and play an important role in plant drought tolerance mechanisms. The seedling stage is very sensitive to drought stress, and it needed to explore the methods and plant characteristics that contribute to drought tolerance in cotton.

**Results:**

Initially, seedlings of 18 genotypes from three *Gossypium* species: *G. hirsutum*, *G. barbadense,* and *G. arboreum,* were evaluated for various seedling traits under control (NS) and drought stress (DS). Afterward, six genotypes, including two of each species, one tolerant and one susceptible, were identified based on the cumulative drought sensitivity response index (CDSRI). Finally, growth rates (GR) were examined for shoot and root growth parameters under control and DS in experimental hydroponic conditions. A significant variation of drought stress responses was observed across tested genotypes and species. CDSRI allowed here to identify the drought-sensitive and drought-resistant cultivar of each investigated species. Association among root and shoots growth traits disclosed influential effects of enduring the growth under DS. The traits including root length, volume, and root number were the best indicators with significantly higher differential responses in the tolerant genotypes. These root growth traits, coupled with the accumulation of photosynthates and proline, were also the key indicators of the resistance to drought stress.

**Conclusion:**

Tolerant genotypes have advanced growth rates and the capacity to cop with drought stress by encouraging characteristics, including root differential growth traits coupled with physiological traits such as chlorophyll and proline contents. Tolerant and elite genotypes of *G. hirsutum* were more tolerant of drought stress than obsolete genotypes of *G. barbadense* and *G. arboreum*. Identified genotypes have a strong genetic basis of drought tolerance, which can be used in cotton breeding programs.

**Supplementary Information:**

The online version contains supplementary material available at 10.1186/s12870-022-03724-4.

## Background

Cotton is the source of natural fiber and contributes 35% of the total global fiber needs. Warmer and dry climates are the major growing areas of cotton, so unconditionally, it exposes to drought stress at various critical growth stages throughout the growth period [1]. Water availability is the key factor for any crop production system. However, the water-scarcity conditions seriously threaten sustainable cotton production [2]. Thus, drought stress is the most deleterious factor limiting plant growth and final crop yield in cotton [3]. During the development, growth, and reproduction stages, plants illustrate more vulnerable behavior to abiotic stresses. Drought stress is the major factor limiting crop productivity and substantially reducing final yield. Cotton has evolved different morpho-physiological and biochemical mechanisms to overcome drought stress [4].

Different plant traits might be the key indicators of how plants tolerate drought stress. Drought stress can affect seedling growth, including shoot length, leaf size, leaf area, and dry leaf weight [5]. The decline in several physiological traits, including photosynthesis and chlorophyll contents, has also been observed as plant water stress increases [6]. It has been argued that the decline in photosynthesis is primarily due to the leaf relative water constants (RWC) [7], and stomatal limitation also has been observed [5, 8, 9]. Additionally, roots are the sensors of plants and detect osmotic stress, possibly altering the physiological and water potential status. Hence, they can play a role in the plant drought tolerance mechanism [10]. Thus, varying moisture conditions and soil temperature are the major abiotic stress factors defining the seedling establishment and early root growth for arid and semi-arid areas over much of the cotton-growing belt globally.

Different root systems have been reported in plants. The plants with deep root systems show better drought tolerance and improve water and nitrogen uptake. Long roots reduce axial roots and lateral branching, which reduces the metabolic cost and maintains plant metabolic activity under drought stress [11]. Drought tolerance at the seedling growth stage in cotton is closely associated with root morphology [12–14]. Pace et al. (1999) also observed a significant increase in root thickness and root length in cotton seedlings under drought stress than control plant seedlings after the drought recovery behavior of cotton. Meanwhile, the increase in chlorophyll contents and RWC help to adjust the stomatal closure approach and accumulate more water contents in the leaf tissue to uphold the photosynthetic activities [15]. Accumulation of proline contents in shoot and root confine cell death by maintaining osmotic adjustment in leaves and roots [10, 16]. Various seedling traits were studied under drought stress, including root length, shoot length, leaf area, number of lateral roots, excised leaf water loss, relative water content, stomatal density, and stomatal conductance are significantly affected. Additionally, the correlation was also revealed a positive and significant association between most of these traits [17].

Various studies have been reported that *G. hirsutum* [18], *G. barbadense* [19], and *G. arboreum* [2] are sensitive to abiotic stress conditions. Different cotton species are well known for possessing additional favorable attributes for cotton yield and adaptability. *G. barbadense* L. has extralong fiber [20], and *G. arboreum* L. has more biotic and abiotic stress tolerance with more adaptability to arid and semi-arid conditions [2, 21]. However, the comparative exploration of seedling responses of different *Gossypium* species under drought stress is still missing.

Various approaches have been employed to explore the genetic potential and performance of cotton genotypes at seedling stages under drought stress among cotton genotypes, including morphological characteristics [14], root growth traits [22], cotton seedling traits [23], electrolyte leakage [24], seed germination characteristics [25], and relative growth rate (RGR) [7]. These incredible approaches could be employed to select and develop drought-tolerant cotton varieties with improved root growth and seedling vigor under drought stress conditions. However, basic knowledge concerning the potential of drought tolerance in cotton is available. Based on previous reports and studies, we hypothesized that early seedling vigor and root growth traits might have a strong association and be the most important characteristics for healthy seedlings, assisting the plant facing drought stress with limited yield losses.

One of the major objectives of this comprehensive study was to quantify the differential responses and variation of species and genotypes to PEG-induced drought stress among the three cotton species and 18 genotypes. The definite objective of this study was to determine which shoot and growth parameters are the most important predictors of seedling drought tolerance in cotton.

## Results

### Variability of drought stress response indices in cotton seedlings

In the screening experiment, 18 genotypes of three cotton species, including *G. hirsutum* (*Gh*), *G. barbadense* (*Gb*), and *G. arboreum* (*Ga*) were subjected to evaluation at early seedling stages under control (NS) and PEG induced drought stress (DS) (Supplementary Table S2). We calculate drought stress response indices (DSRI) of individual genotype for each trait to observe the comparative response among the genotypes and species (Supplementary Table S3). Hierarchical clusters and graphical plots were constructed to categorize the genotypes into different groups based on DSRI values of 18 cotton genotypes for 19 seedling traits. A multivariate, principal component, and biplot analysis (PCA) was performed to investigate the response, the variability, and association among seedling traits of cotton genotypes toward PEG-induced drought stress.

### Hierarchical cluster analysis of drought stress response indices (DSRI)

**Fig. 1:**
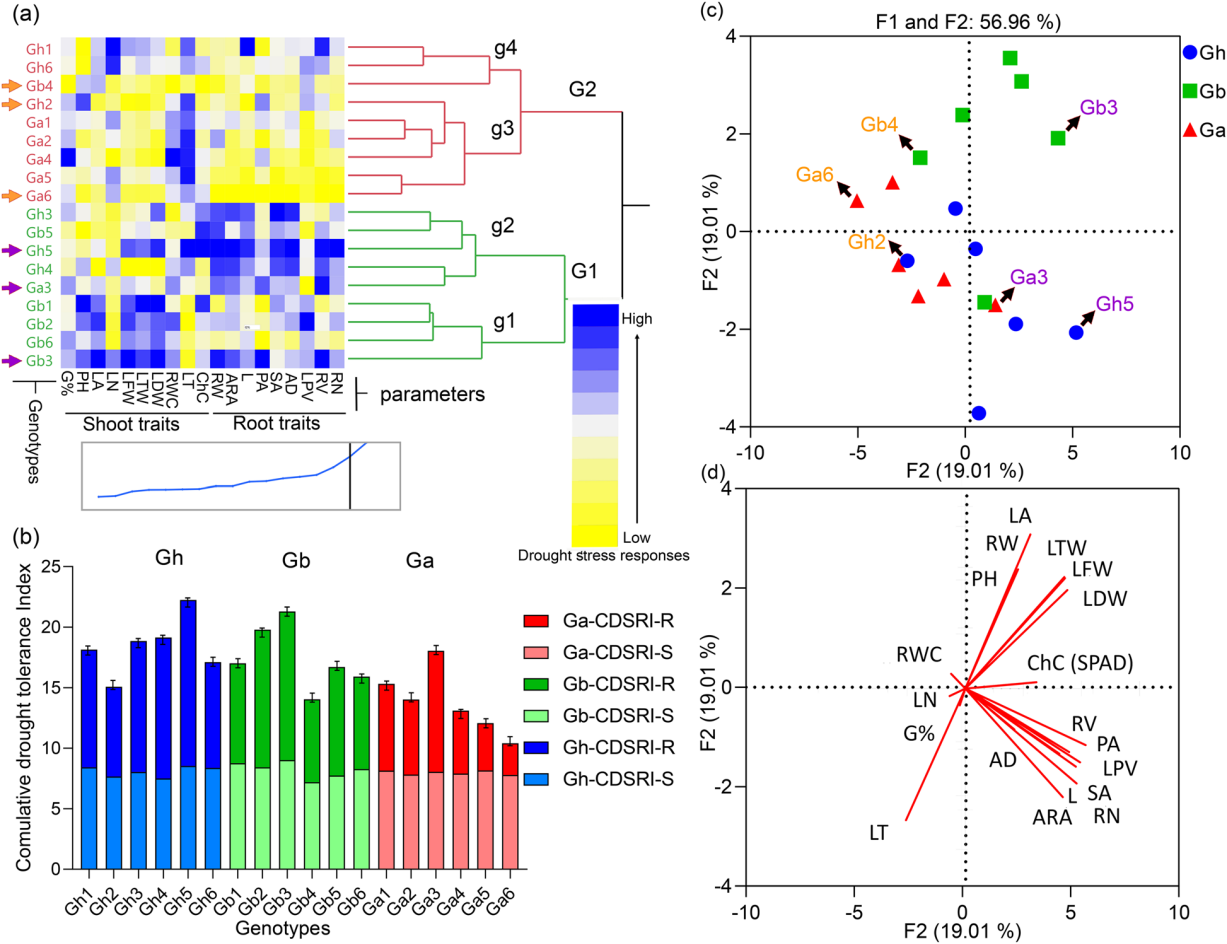
Drought stress responses and variation among a panel of 18 cotton genotypes, including six from each three-cotton species *G. hirsutum*, *G. barbadense,* and *G. arboreum*. **a** Hierarchical Cluster and Dendrogram, genotypes are distributed in two major (G1 and G2) and four sub-groups (g1, g2, g3, and g4). **b** Graphs of cumulative drought stress response index (CDSRI) including cumulative drought stress response index of shoot traits (CDSRI-S) and root traits (CDSRI-R) for 18 genotypes of three cotton species. **c** groups. **c** & **d** Biplot (Principal component analysis) of drought stress response indices (DSRI) for morpho-physiological and early root growth traits at the seedling stage. Scatter-plot shows the distribution of identified drought tolerant (purple) and susceptible (orang) cotton genotypes. Here PH, plant height; ChC, chlorophyll contents; LN, leaf number; LA, leaf area; LT leaf temperature; LFW, leaf fresh weight; LDW, leaf dry weight; LDW, leaf turgid weight; RWC, relative water contents; RL, root length; ARA, analyzed region area; ARW analyzed region width; ARH, analyzed region height; SA surface area; LPA, root length per area; AD average diameter; RV, root volume; RH, root heigh; RN root number; RF root forks; RC root crosses; RW root weight and R/S, root to shoot ratio

We construct the hierarchical cluster and graphical comparison analysis of drought stress responses indices in 18 cotton genotypes to identify the drought-tolerant and susceptible genotypes and investigate the variation among the genotypes and traits (Fig. 1). There were two major groups (G1 and G2) containing nine genotypes in each group and four sub-groups (g1, g2, g3, and g4). Among the sub-groups, g3 had 6, a maximum number of genotypes including 5 of *Ga* and 1 of *Gh* genotypes. g4 had a minimum of 3 genotypes, including Gh1, Gh6, and Gb4. In G1, g1 and g2 had significantly higher DSRI for most of the traits, g1 had higher DSRI for shoot growth traits, and g2 had higher DSRI for most root growth traits. Meanwhile, genotypes in g3 and g4 had less response to drought stress and were identified as drought-susceptible genotypes. Major groups G1 and G2 had significant distention, which helps classify the genotypes as susceptible and tolerant (Fig. 1a).

Graphical comparison of CDSRI showed a clearer response of genotypes for drought stress, which indicates that Gh5, Gb3, and Ga3 are the most drought-tolerant genotypes, while Gh2, Gb4, and Ga6 stand drought susceptible genotypes among the studied genotypes. Interestingly, tolerant genotype had more contribution of CDSRI-R than CDSRI-S, and CDSRI-S also had less variation, especially in *G. arboreum* (Fig. 1b). These analyses portray a clearer distribution of genotypes in different groups based on the drought stress response of studied traits. The results showed significant variability in drought stress responses of studied genotypes and differential response to the NS and DS conditions.

### Principal component & biplot (PCA) and correlation analysis

The distribution of genotypes and traits in the PCA biplot explained the high variability of traits for principal components F1 and F2. In PCA of all 18 genotypes, *Gb* genotypes had a significantly higher response for shoot traits, while *Gh* genotypes had a higher response for root traits. In contrast, *Ga* genotypes had comparatively less response for both sets of traits (Fig. 1c and d). The genotypes of three different species are distributed in different groups in the PCA biplot (Fig. 1c). Traits vectors also explain two distinct clusters of shoot root traits in the PCA biplot (Fig. 1d). Genotypes in purple color have been identified as drought-tolerant genotypes, while in blue color have been identified as susceptible genotypes for further root growth studies.

While separately plotted biplots of *Gh*, *Gb*, and *Ga* indicating overall variability of 67.2%, 72.9%, and 78.0%, respectively (Supplementary Figure S1a). Vectors of PH and LA in *Gh*, RV, ARA in Gb, and G% and RWC in Ga had higher magnitude, indicating the high variability of these traits in the three species. In contrast, RWC had very low variability in *Gh* and *Gb* compared to *Ga*. Root growth parameters including RL, RV, RLV, RN, SA, and ARA, except RW, had a strong association with each other in all three species (Supplementary Figure S1b).

In correlation, RW had significant associations with shoot growth traits such as LN and LA in *Gh*, PH, and leaf weight parameters. Interestingly, ChC had a strong positive association with root growth parameters in *Gh* and *Gb* except for *Ga*. In *G. hirsutum,* Gh5 has shown a significant response towards ChC, RN, SA LDW, and many other traits, but Gh2 showed a negative response towards most traits and fell away from most of the traits in the biplot. Among the *G. barbadense* genotypes, Gb3 has demonstrated a significant positive response towards LN, RN, RWC, G% PA, and most of the other traits. Meanwhile, Gb4 had a negative response for most of the studied traits and fell away from them in the biplot. In *G. arboreum,* Ga3 had more attraction towards RV, PA, LPV SA, RN, and other root growth parameters. Sametime, Ga6 had repellant behavior to most traits in biplot distributions of genotypes.

### Correlation between shoot and root growth traits

Correlation heat map of shoot and root growth traits have shown in Supplementary Figure S1b under control (NS) and PEG-induced drought stress (DS) conditions. Plant height significantly correlated with leaf weight parameters in shoot growth traits, including LA, LDW, LTW, RWC, LT, and root growth parameters, including AD and RV. At the same time, PH negatively correlated with LN, ChC, and RW under NS conditions. Meanwhile, PH had a significant positive correlation with root growth parameters under DS, including RW, RA, SA, AD, and RV. Relative water contents had a significant negative correlation with ChC, RW, LPV under NS conditions, while under DS it had a positive correlation with these traits, which indicates the role of RWC in drought tolerance and root growth under DS conditions. Leaf growth traits had a more positive and strong correlation with root growth traits under DS conditions than under NS conditions. Likewise, root growth traits had a weaker association among each other under NS conditions than under DS conditions, which clearly indicates the role of these traits in supporting each other under DS conditions. Meanwhile, the correlation among the drought stress response indices of growth rate (DSRI-GR) has shown in Supplementary Figure S2.

### Growth rates variability in drought-tolerant and susceptible genotypes under drought stress (DS)

**Fig. 2:**
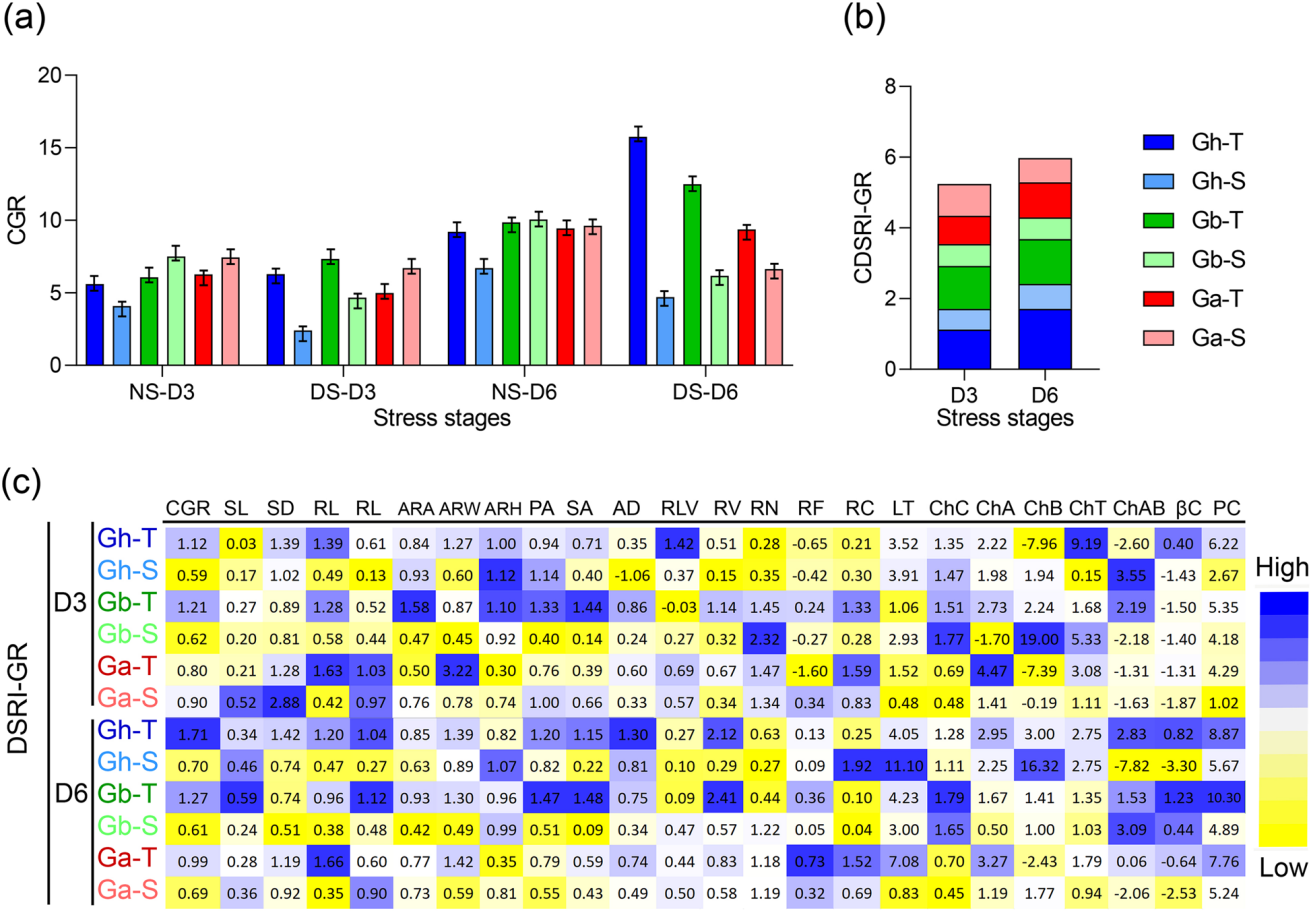
Cumulative growth rate (CGR) and cumulative drought stress response indices of growth rate (DSRI-GR) of 24 shoot and root growth traits of six cotton genotypes, including two genotypes of each three species, one drought-tolerant and one drought susceptible genotype grown in hydroponic conditions under non-stress (NS) PEG-induced drought stress (DS) conditions. **a** CGR comparison of six genotypes; **b** proportional comparison of DSRI-GR of six cotton genotypes; **c** Heatmap of CGR and GR for all traits. Mean values of all traits have given in (Supplementary Table 4). Here SL, shoot length; SD, stem diameter; LT, leaf temperature; ChC, chlorophyll contents (SPAD); ChA, chlorophyll A; ChB chlorophyll B; ChT, total chlorophyll; A/B, chlorophyll A/B ratio; BC, β-carotenoids, PC, proline contents; RL, root length; ARA, analyzed region area; ARW analyzed region width; ARH, analyzed region height; SA surface area; LPA, root length per area; AD average diameter; RV, root volume; RH, root heigh; RN, root number; RF, root forks; RC root crosses; RW root weight; R/S, root to shoot ratio; D3, three days after stress; D6, 6 days after stress

After performing a screening experiment of 18 genotypes of three cotton species, including *G. hirsutum*, *G. barbadense,* and *G. arboreum*, we identified six cotton genotypes, including two genotypes; one drought-tolerant and one drought susceptible genotype of each three species. The selected tolerant and susceptible genotypes were re-named for easy comparison: *G. hirsutum*, Gh6 to Gh5-T (tolerant), Gh2 to Gh-S (susceptible); *G. barbadense*, Gb3 to Gb-T (tolerant), Gb4 to Gb-S (susceptible); and *G. arboreum*, Ga3 to Ga-T (tolerant) Ga6 to Ga-S (susceptible). These genotypes were subjected to evaluation for growth rates (GR) at early seedling stages after three days (D3) and six days of stress under control (NS) and PEG-induced drought stress (DS) (Supplementary table S5). A cumulative growth rate (CGR) comparison was made among each species and all genotypes (Fig. 2a). We also calculated drought stress response indices for each trait's growth rate (DSRI-GR) of individual genotypes to observe the comparative response among the genotypes and species. DSRI-GR heatmap of 24 shoot and root growth traits of six cotton genotypes was constructed to determine the overall growth performance of contrasting genotypes under NS and DS conditions (Fig. 2b and c).

In the overall proportional comparison of six genotypes (Fig. 2b), Gh-T and Gb-T Ga- had comparatively higher CDSRI-GR of 1.2 and 1.21 compared to susceptible three genotypes at D3. While Gh-T, Gb-T, and Ga-T had enhanced CDSRI-GR of 1.7, 1.27, and 0.99, respectively, at D6. Gh-T had an 8% gain in CDSRI-GR at D6 compared to D3, but Gb-T and Ga-T just had a 1% increase among all six genotypes. This indicates that Gh-T had the highest comparative GR index among all the genotypes. Meanwhile, Ga-S had the comparatively lowest CDSRI-GR at D6 among all genotypes.

## Discussions

### Effect of drought stress on cotton seedlings

Vigorous plant seedlings have an important role in crop yield performance, but various abiotic stresses consistently affect plant growth by distressing their normal growth at the early growth stages [26]. A considerable variation was observed for various morpho-physiological traits in studied genotypes, and the genotypes clearly fell into two groups, G1 and G2. The genotypes of group G2 are considered drought-tolerant with significantly higher values for most of the shoot and root traits, proving them drought-tolerant genotypes. Meanwhile, the genotypes of group G2 are considered drought-susceptible, with significantly lower values for most of the shoot and root traits (Fig. 1a).

**Fig. 3:**
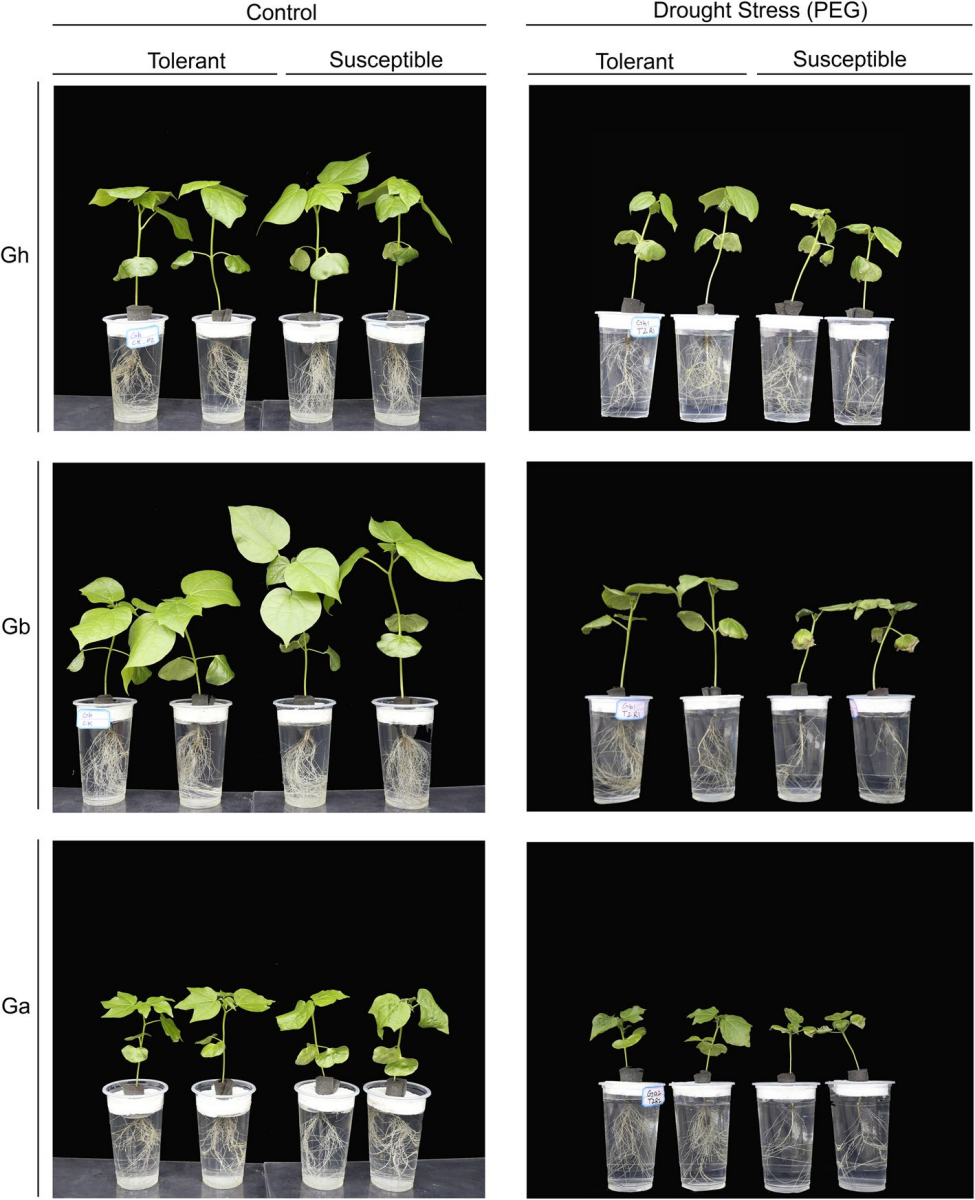
Comparison of the cotton shoot and root growth of three cotton species under control (NS) and PEG-induced drought stress (DS) at early growth stages

Reduction in plant biomass, including shoot and root growth traits, was observed under the DS conditions, indicating less assimilation and reduction in the uptake of nutrients by the roots due to the low water potential cultivated under osmotic stress. Meanwhile, such nutrition imbalances caused the stunted growth of plant seedlings by enhancing the nutrient utilization compared to nutrient uptake efficiency and energy resources, which are the reasons for the reduction in plant biomass [27], including shoot and root weights (Fig. 3). Drought stress causes a decrease in various morpho-physiological traits that have been well reported previously [18].

An increase in chlorophyll content was observed in drought-tolerant genotypes. Increased RWC was also noticed under DS conditions, which depicts that tolerant genotypes adjust the stomatal closure approach and accumulate more water contents in the leaf tissue to uphold the photosynthetic activities [15]. Drought stress conditions rapidly decline chlorophyll A, B, and total contents, ultimately reducing photosynthate production [28] and restricting plant growth. The low value of the ChA/B ratio is also a solid indicator of selecting tolerant genotypes under abiotic stress conditions (Ali et al., 2009). It was observed that tolerant genotypes have higher photosynthesis and RWC contents than susceptible genotypes [28]. Accumulation of proline contents in drought-tolerant genotypes was noticed, which probably reduces cell death by maintaining osmotic adjustment in plant leaves and roots [10, 16].

Previous studies [12, 14, 29] are consistent with our results that shoot and root growth are significantly affected by drought and other abiotic stresses. Genetic variability among the genotypes for root growth traits such as root length and root area under DS condition supports the conclusions of Singh et al. and Basal et al. [12, 14]. The root growth traits are important characters influencing shoot growth and seedling vigor in cotton under DS conditions. Furthermore, the higher root diameter and area under stress conditions than NS agreed with [14, 30]. They reported higher root diameter in corn [30] and cotton [14] seedlings under DS than control. Singh et al. (2018) also reported high genetic variability for various root growth traits among nine elite cotton genotypes under drought and cold stress. He explained that greater root growth might be attributed to greater xylem size and root thickness under stress, leading to longer roots, deep penetration, and increased water uptake [14]. A significantly improved performance was observed in modern *G. hirsutum* genotypes compared to obsolete cotton genotypes of *G. barbadense* and *G. arboreum*. It was explained recently that modern cotton cultivars [31] and elite cotton lines [14] had improved performance than obsolete cotton cultivars in *G. hirsutum* under drought stress conditions.

A significant increase in growth rate (GR) of some shoot traits and several root growth traits under DS conditions indicates the differential response of genotypes to counter the destruction of drought stress at early seedling growth stages in cotton. An increase in root length, root area, root numbers, and root volume was observed, which helped the plant seedlings to cope with the drought stress effects and resume the shoot growth. A strong positive association of SL with several root growth traits is evidence of this growth behavior of cotton seedlings, as previously reported [14, 29, 32]. The positive association between root growth traits and physiological shoot traits, including ChC, ChA/B ratio [33, 34], and PC [10], also indicates the significant effects of enduring the growth under stress conditions. We identified drought-tolerant genotypes including Gh-T, Gb-T, and Ga-T had higher GRs for most of the traits than drought susceptible genotypes under DS conditions. It indicates the incredible role of root growth in sustaining seedling growth, which also specifies the differential response and development of roots to observe more water and nutrients to overcome the shoot growth losses [29, 35, 36].

### Responses of shoot and root growth rates to drought stress

Under NS conditions, growth rate measures at D3 (three days after stress) and D6 (6 days after stress) had less significant differences among all traits with few exceptions. Meanwhile, it significantly increased root growth traits under DS conditions at both D3 and D6, indicating differential root growth under drought stress. Root length, volume, and root number significantly increased in drought-tolerant genotypes [32, 37]. The different response of root growth parameters among three species was observed, such as in *G. arboreum*, the GR of root length was higher than the root volume and root surface area. On the other hand, in *G. hirsutum* and *G. barbadense,* the GR of root volume and root surface were simultaneously increased with root length (Fig. 2). It indicates that different species have different mechanisms and approaches of root growth to counter the drought stress and respond differently under stress conditions. Recently, it has been reported that cotton species have different mechanisms against drought stress [38]. In *G. hirsutum*, GR of shoot traits including ChC, ChA, ChB, ChT, and PC, along with root traits, had maximum increase under DS compared to other species, which also aligned with previous studies [10, 33, 34]. It also explained that elite cotton cultivars proved more drought tolerance than obsolete genotypes [14, 31]. So, it might be the reason for high tolerance in *G. hirsutum* genotype among the studied genotypes. An increase in root growth assists plants in uptake more nutrients and water to maintain the photosynthetic activities and counter the drought stress effects [35], which ultimately restore the shoot growth and increase the assimilation of nutrients in the upper parts of the plant [14, 39].

A single value of cumulative drought stress response index of growth rate (CDSRI-GR) for a genotype provides us an opportunity to compare the performance and growth rate of genotypes and species among each other. Our results revealed that drought-tolerant genotypes had significantly higher CDSRI-GR than susceptible genotypes in all three species measured at D6. This indicates that tolerant genotypes can maintain growth under drought stress and even have higher growth rates of root growth traits depicting that root growth gets promoted in tolerant genotypes under stress [39]. Among the species, In *G. hirsutum, G. barbadense* had higher CGR and CDSRI-GR than *G. arboreum* at D3. At the same time, *G. arboreum* showed a higher increase in CGR and CDSRI-GR at D6, which indicates the quick response to drought stress in *G. hirsutum G. barbadense* than *G. arboreum*. Previously, it was also explained by Xu et al. (2010).

## Conclusion

Drought stress can cause a considerable reduction in seedling emergence rate, followed by poor seedling vigor in arid and semi-arid areas. The studied germplasm had credible variability for all the observed traits, with some exceptions. Association among root and shot growth traits have significant effects of enduring stress conditions. Root length, volume, and number indicated a significantly higher differential response to drought stress in drought-tolerant genotypes. Tolerant genotypes can advance growth rate, maintain growth under drought stress, and encourage root growth in tolerant genotypes under stress. Drought tolerant and advanced genotypes of *G. hirsutum* explained more drought tolerance than obsolete genotypes of *G. barbadense* and *G. arboreum*. Among the studied traits, including chlorophyll and proline contents, the root length, volume, and number are the potential traits that can be used to screen the drought-tolerant material or varieties of cotton. The selection criteria of drought stress response indices and growth rate also prove reliable and could be exploited to develop stress residence crop varieties. Identified three cotton species genotypes can be explored as genetic resources for drought tolerance in cotton breeding programs.

## Material and methods

### Plant material

A panel of 18 cotton genotypes, genetically diverse, was selected from the previously Characterized, genotyped, and screened germplasm (genotypes) of three cotton species (Spp.), including; 420 *Gossypium hirsutum* (*Gh*) genotypes [40], 360 *Gossypium barbadense* (*Gb*) genotypes [41], and 215 *Gossypium arboreum* (*Ga*) genotypes [42] (genotype IDs are mentioned in Supplementary Table S1). Six genotypes from each Spp., including three drought-tolerant and three drought susceptible genotypes, were selected based on drought tolerance indices (unpublished data). The pure seeds of genotypes were collected from the Cotton Germplasm Recourses, Cotton Research Institute of Chinese Academy of Agricultural Sciences (ICR, CAAS), Anyang, Henan, China. The experiments were conducted at the growth room facility of the State Key Laboratory of Cotton Biology, ICR, CAAS. The accessions are genotyped and characterized as distinct genotypes,

#### Experimental conditions for the first seedling experiment

Seeds of 18 cotton genotypes were grown in specially designed plastic cubes for seedling growth (Supplementary Figure S3), filled with the mixture of peat-media and vermiculite in a 3:1 ratio, at two moisture levels; control at 100% filed capacity (NS) and drought stress at 50% filed capacity (DS) in the growth room. A 15% concentration of polymethyl glycol (PEG 6000) was used to apply the drought stress 12 days after sowing. Five plants of each genotype were maintained in four replications under a complete randomized design (CRD). The growth room conditions were maintained at photoperiod: 16/8 h light/dark with 2200-2400 lx, temperature: at 30 °C ± 2 (light)/23 °C ± 2 (dark), and relative humidity: 60–65% through an automatic system. Three weeks after the application of DS treatment, data were collected for the morphological and physiological traits, including plant height (PH), chlorophyll contents (ChC), leaf number (LN), leaf area (LA), leaf temperature (LT), leaf fresh weight (LFW), leaf dry weight (LDW), leaf turgid weight (LDW), and relative water contents (RWC).

A separate experiment was conducted to measure the root traits in germination pouches (www.phytotc.com) (Supplementary Figure S4). Ten seeds of each genotype were planted in germination pouches in three replications after disinfecting the seed surface to avoid fungal growth. Initially, 80 ml dd.H_2_O was applied to control (NS), and 80 ml of PEG solution with 10% concentration was applied to impose the drought stress (DS); the treatment application was repeated after one week. After two weeks of planting, the roots were scanned with an HP scanner at 600 dpi in an A4 size transparent tray filled with water. Subsequently, the roots were analyzed with WinRHIZO software (2016 pro) (Regent Instruments, Inc., QC, Canada) to measure the root morphological traits, including root length (RL), analyzed region area (ARA), analyzed region width (ARW), analyzed region height (ARH), surface area (SA), root length per area (LPA), the average diameter (AD), root volume (RV), root height (RH), root number (RN), root forks (RF), and root crosses. We also calculated the root weight (RW) and root to shoot ratio (R/S).

#### Data measurement and experimental conditions for the hydroponic experiment

The selected tolerant and susceptible genotypes were re-named for easy comparison: *G. hirsutum*, Gh6 to Gh5-T (tolerant), Gh2 to Gh-S (susceptible); *G. barbadense*, Gb3 to Gb-T (tolerant), Gb4 to Gb-S (susceptible); and *G. arboreum*, Ga3 to Ga-T (tolerant) Ga6 to Ga-S (susceptible). The seeds of six identified genotypes were disinfected with 75% ethanol and 0.1% HgCl_2_ solution. Subsequently, the seeds were germinated in the seedling plate for 5–6 days. The uniform shoot and root size seedlings were transplanted to a container of 15 L volume filled with half (1/2) strength Hoagland solution. After one week, the full-strength Hoagland solution was applied for subsequent applications. The solution media was continuously aerated and refreshed after every five days. The experiment was divided into control and drought-stressed sets, with three replications having five plants in each replication. 8% PEG solution was applied after one week of seedling transfer in the hydroponic media. Data of shoot growth traits including shoot length (SL), stem diameter (SD), leaf temperature (LT), SPAD chlorophyll contents (ChC), chlorophyll A (ChA), chlorophyll B (ChB), total chlorophyll (ChT), chlorophyll A/B ratio (ChA/B), β-carotenoids (BC), proline contents (PC) and root growth traits were the same as measured in the first experiment. After one week of transfer of the seedlings in hydroponic media, the data was measured at three different times, including zero-days of stress (D0), after three days of stress (D3), and after six days of stress (D6) of both NS and DS set of plants.

Among the physiological traits, relative water content (RWC) by [43], proline content by [44], and chlorophyll contents, including chlorophyll a, b, and total chlorophyll, were estimated by the following equations proposed by (Nippon, 1992).

$$\mathrm{Chlorophyll a }(\mathrm{mg}/100\mathrm{mL}) = 0.999\mathrm{A}663 - 0.0989\mathrm{A}645$$

$$\mathrm{Chlorophyll b }(\mathrm{mg}/100\mathrm{mL}) = -0.328\mathrm{A}663 + 1.77\mathrm{A}645$$

(A453, A505, A645, and A663 are absorbance at 453 nm, 505 nm, 645 nm, and 663 nm, respectively).

### Drought stress response index

Cotton genotypes were clustered based on their degree of drought tolerance, using the drought stress response index (DSRI) described by Wijewardana et al. in corn, and by Singh et al., in cotton [14, 45]. Initially, drought stress response indices for each genotype were estimated by dividing the value obtained under DS by the value under NS for each parameter (Eq. 1). Finally, the cumulative drought sensitivity response index (CDSRI) was calculated individually for each genotype by summing up DSRI, respectively (Eq. 2). Accordingly, cotton genotypes were classified as drought tolerant and susceptible genotypes.

1 $$\mathrm{DSRI }(\mathrm{individual trait}) =\mathrm{ value under DS}/\mathrm{value under NS}$$

2 $$\mathrm{CDSRI}=\mathrm{ DSRI}\_1 +\mathrm{ DSRI}\_2+\dots \mathrm{ DSRI}-\mathrm{n}$$

Here, 1, 2, and n are the traits from 1 to nth number.

### Growth rate and drought stress response index of growth rate

Identified and selected genotypes based on CDSRI were subjected to evaluation for growth rates (GR) for individual traits by the following equation (Eqs. 3 and 4) for three days after stress (D3) and six days after stress (D6). We use a modified equation [7] to calculate the relative growth rate in cotton seedlings. The individual trait growth rate was calculated separately under control (NS) and PEG-induced drought stress (DS) conditions. We also computed drought stress response indices for growth rate (DSRI-GR) of individual traits for D3 and D6 (Eqs. 5 and 6). Finally, the cumulative drought sensitivity response index for growth rate (CDSRI-GR) was calculated individually for each genotype by summing up DSRI-GR for D3 and D6, respectively (Eqs. 7 and 8).

3 $$\mathrm{GR }(\mathrm{after }3\mathrm{ days}) =\mathrm{ value at D}3 -\mathrm{ value at D}0 /\mathrm{ value at D}3$$

4 $$\mathrm{GR }(\mathrm{after }6\mathrm{ days}) =\mathrm{ value at D}6 -\mathrm{ value at D}0 /\mathrm{ value at D}6$$

5 $$\mathrm{DSRI}-\mathrm{GR }(\mathrm{after }3\mathrm{ days}) =\mathrm{ GR}(\mathrm{D}3)\mathrm{ under DS}/\mathrm{ GR}(\mathrm{D}3)\mathrm{ under NS}$$

6 $$\mathrm{DSRI}-\mathrm{GR }(\mathrm{after }6\mathrm{ days}) =\mathrm{ GR}(\mathrm{D}6)\mathrm{ under DS}/\mathrm{ GR}(\mathrm{D}6)\mathrm{ under NS}$$

7 $$\mathrm{CDSRI}-\mathrm{GR }(\mathrm{after }3\mathrm{ days}) =\mathrm{ DSRI}-\mathrm{GR}\_1 +\mathrm{ DSRI}-\mathrm{GR}\_2+\dots \mathrm{ DSRI}-\mathrm{GR}\_\mathrm{n}$$

8 $$\mathrm{CDSRI}-\mathrm{GR }(\mathrm{after }6\mathrm{ days}) =\mathrm{ DSRI}-\mathrm{GR}\_1 +\mathrm{ DSRI}-\mathrm{GR}\_2+\dots \mathrm{ DSRI}-\mathrm{GR}\_\mathrm{n}$$

Here, 1, 2, and n are the traits from 1 to nth number

### Statistical analysis

Analysis of variance (ANOVA) was constructed to test the effects of treatments, genotypes, and genotypes into treatment interaction. Hierarchical clustering, dendrogram, and constellation plot were constructed by the ward method. JMP®, Version 15 carried out principal component analysis (PCA) biplot and correlation matrix. SAS Institute Inc., Cary, NC, 1989–2019.

## Supplementary Information

**Additional file 1: Supplementary Figure S1.** Biplot (Principal component analysis) of drought stress response indices (DSRI) for morpho-physiological and early root growth traits at the seedling stage. Scatter-plot shows the distribution of identified drought tolerant (purple) and susceptible (orang) cotton genotypes in (a). Correlation matrix heat map of shoot growth traits and root growth traits under NS (left) and DS (right) conditions in (b). Here PH, plant height; ChC, chlorophyll contents; LN, leaf number; LA, leaf area; LT leaf temperature; LFW, leaf fresh weight; LDW, leaf dry weight; LDW, leaf turgid weight; RWC, relative water contents; RL, root length; ARA, analyzed region area; ARW analyzed region width; ARH, analyzed region height; SA surface area; LPA, root length per area; AD average diameter; RV, root volume; RH, root heigh; RN root number; RF root forks; RC root crosses; RW root weight and R/S, root to shoot ratio. **Supplementary Figure S2.** Correlation among the drought stress response indices of growth rate (DSRI-GR) for 24 shoot and root growth traits in 6 cotton genotypes. **Supplementary Figure S3.** 18 cotton accessions sown in plastic cubes for shoot growth traits. **Supplementary Figure S4.** 18 cotton accessions sown in germination pouches for root growth traits. **Supplementary Figure S5.** Selected six cotton accessions sown in hydroponic condition for shoot and root growth traits. **Supplementary Table S1.** Details of accessions used in this study. **Supplementary Table S2.** Analysis of variance (ANOVA) of 18 cotton accession of three species including *G. hirsutum*, *G. barbadense,* and *G. arboreum, *for 18 shoot and root growth traits under control (NS) and PEG-induced drought stress (DS). **Supplementary Table S3.** Drought stress response index (DSRI) and cumulative drought stress response index (CDRI) of 18 cotton genotypes, including 6 genotypes from each three-cotton species *Gossypium hirsutum*, *Gossypium barbadense,* and *Gossypium arboreum*. **Supplementary Table S4.** Mean values of the shoot and root growth traits were measured at three different times, including zero days of stress (D0), after three days of stress (D3), and after six days of stress (D6) under NS DS conditions. **Supplementary Table S5.** Growth rate (GR) of indivisual shoot and root growth traits were measured at three different times, including zero days of stress (D0), after three days of stress (D3), and after six days of stress (D6) under NS DS conditions.

## Data Availability

The data sets used and analyzed during the present study are included in this published article and its supplementary information files. Study protocol comply with relevant institutional national and international guidelines and legislation. We have permission to use the plant material for experimental purposes.

## References

[1] Abdelraheem A, Esmaeili N, O’Connell M, Zhang J (2019). Progress and perspective on drought and salt stress tolerance in cotton. Ind Crops Prod.

[2] Iqbal M, Khan MA, Chattha WS, Abdullah K, Majeed A (2019). Comparative evaluation of Gossypium arboreum L. and Gossypium hirsutum L. genotypes for drought tolerance. Plant Genet Resour Characterisation Util..

[3] Mahmood T, Khalid S, Abdullah M, Ahmed Z, Shah MKN, Ghafoor A (2020). Insights into drought stress signaling in plants and the molecular genetic basis of cotton drought tolerance. Cells.

[4] Mahmood T, Wang X, Ahmar S, Abdullah M, Iqbal MS, Rana RM (2021). Genetic potential and inheritance pattern of phenological growth and drought tolerance in cotton ( Gossypium Hirsutum L. ). Front Plant Sci..

[5] Pettigrew WT (2004). Moisture deficit effects on cotton lint yield, yield components, and boll distribution. Agron J.

[6] Chastain DR, Snider JL, Choinski JS, Collins GD, Perry CD, Whitaker J (2016). Leaf ontogeny strongly influences photosynthetic tolerance to drought and high temperature in Gossypium hirsutum. J Plant Physiol.

[7] Sekmen AH, Ozgur R, Uzilday B, Turkan I (2014). Reactive oxygen species scavenging capacities of cotton (Gossypium hirsutum) cultivars under combined drought and heat induced oxidative stress. Environ Exp Bot.

[8] Lawlor DW, Cornic G (2002). Photosynthetic carbon assimilation and associated metabolism in relation to water deficits in higher plants. Plant, Cell Environ.

[9] Chastain DR, Snider JL, Collins GD, Perry CD, Whitaker J, Byrd SA (2014). Water deficit in field-grown Gossypium hirsutum primarily limits net photosynthesis by decreasing stomatal conductance, increasing photorespiration, and increasing the ratio of dark respiration to gross photosynthesis. J Plant Physiol.

[10] Zhang L, Peng JT, Chen TH, Zhao XP, Zhang SD, Liu S (2014). Effect of drought stress on lipid peroxidation and proline content in cotton roots. J Anim Plant Sci..

[11] Lynch JP (2022). Harnessing root architecture to address global challenges. Plant J.

[12] Basal H, Smith CW, Thaxton PS, Hemphill JK (2005). Seedling drought tolerance in upland cotton. Crop Sci.

[13] Pace PF, Cralle HT, El-Halawany SHM, Cothren JT, Senseman SA (1999). Drought-induced changes in shoot and root growth of young cotton plants. J Cotton Sci.

[14] Singh B, Norvell E, Wijewardana C, Wallace T, Chastain D, Reddy KR (2018). Assessing morphological characteristics of elite cotton lines from different breeding programmes for low temperature and drought tolerance. J Agron Crop Sci.

[15] Chaves MM, Flexas J, Pinheiro C (2009). Photosynthesis under drought and salt stress: regulation mechanisms from whole plant to cell. Ann Bot.

[16] Mahmood T, Abdullah M, Ahmar S, Yasir M, Iqbal MS, Yasir M (2020). Incredible role of osmotic adjustment in grain yield sustainability under water scarcity conditions in wheat (Triticum aestivum L.). Plants..

[17] Veesar NF, Jatoi WA, Channa QA, Memon S, Gandahi N, Aisha G (2021). Evaluation of cotton genotypes for drought tolerance and their correlation study at seedling stage. Fresenius Environ Bull.

[18] Hassan HM, Azhar FM, Khan AA, Basra SMA, Hussain M (2015). Characterization of cotton (Gossypium hirsutum) germplasm for drought tolerance using seedling traits and molecular markers. Int J Agric Biol.

[19] Hamoud H, Soliman Y, Eldemery S, Abdellatif K (2016). Field Performance and Gene Expression of Drought Stress Tolerance in Cotton (Gossypium barbadense L.). Br Biotechnol J..

[20] Liu X, Zhao B, Zheng H-J, Hu Y, Lu G, Yang C-Q (2015). Gossypium barbadense genome sequence provides insight into the evolution of extra-long staple fiber and specialized metabolites. Sci Rep.

[21] Liu D, Guo X, Lin Z, Nie Y, Zhang X (2006). Genetic diversity of asian cotton (gossypium arboreum l.) in china evaluated by microsatellite analysis. Genet Resour Crop Evol..

[22] Abdelraheem A, Hughs SE, Jones DC, Zhang J (2015). Genetic analysis and quantitative trait locus mapping of PEG-induced osmotic stress tolerance in cotton. Plant Breed.

[23] Megha BR, Mummigatti UV, Chimmad VP, Aladakatti YR (2017). Evaluation of hirsutum cotton genotypes for water stress using peg-6000 by slanting glass plate technique. Int J Pure Appl Biosci..

[24] Cottee NS, Tan DKY, Bange MP, Cheetham JA. Simple electrolyte leakage protocols to detect cold tolerance in cotton genotypes. Proc 4th World Cott Res Conf (Lubbock, Texas, 10–14 Sept 2007). 2008.

[25] Singh B, Raja Reddy K, Redoña ED, Walker T (2017). Developing a screening tool for osmotic stress tolerance classification of rice cultivars based on in vitro seed germination. Crop Sci.

[26] Dugasa MT, Cao F, Ibrahim W, Wu F (2019). Differences in physiological and biochemical characteristics in response to single and combined drought and salinity stresses between wheat genotypes differing in salt tolerance. Physiol Plant.

[27] Hu Y, Burucs Z, Schmidhalter U (2006). Short-term effect of drought and salinity on growth and mineral elements in wheat seedlings. J Plant Nutr.

[28] Nyachiro JM, Briggs KG, Hoddinott J, Johnson-Flanagan AM (2001). Chlorophyll content, chlorophyll fluorescence and water deficit in spring wheat. Cereal Res Commun.

[29] Reddy KR, Brand D, Wijewardana C, Gao W (2017). Temperature effects on cotton seedling emergence, growth, and development. Agron J.

[30] Wijewardana C, Henry WB, Gao W, Reddy KR (2016). Interactive effects on CO2, drought, and ultraviolet-B radiation on maize growth and development. J Photochem Photobiol B Biol.

[31] Brand D, Wijewardana C, Gao W, Reddy KR (2016). Interactive effects of carbon dioxide, low temperature, and ultraviolet-B radiation on cotton seedling root and shoot morphology and growth. Front Earth Sci.

[32] Iqbal A, Dong Q, Wang X, Gui H, Zhang H, Zhang X (2020). High nitrogen enhance drought tolerance in cotton through antioxidant enzymatic activities nitrogen metabolism and osmotic adjustment. Plants (Basel).

[33] Manivannan P, Jaleel CA, Sankar B, Kishorekumar A, Somasundaram R, Lakshmanan GMA (2007). Growth, biochemical modifications and proline metabolism in Helianthus annuus L. as induced by drought stress. Colloids Surf B Biointerfaces..

[34] Hewitt SC, Hewitt SC, Korach KS (2003). Evaluation of the effects of drought on cotton plants using characteristics of chlorophyll fluorescence. Dokl Biol Sci.

[35] Riaz M, Farooq J, Sakhawat G, Mahmood A, Sadiq MA, Yaseen M (2013). Genotypic variability for root/shoot parameters under water stress in some advanced lines of cotton (Gossypium hirsutum L.). Genet Mol Res..

[36] Luo HH, Zhang YL, Zhang WF (2016). Effects of water stress and rewatering on photosynthesis, root activity, and yield of cotton with drip irrigation under mulch. Photosynthetica.

[37] Yu H, Chen X, Hong Y-Y, Wang Y, Xu P, Ke S-D (2008). Activated expression of an arabidopsis HD-START protein confers drought tolerance with improved root system and reduced stomatal density. Plant Cell Online.

[38] Hasan M, Ma F, Islam F, Sajid M (2019). Comparative transcriptomic analysis of biological process and key pathway in three cotton ( Gossypium spp .) species under drought stress. Int J Mol Sci..

[39] Xu Z, Zhou G, Shimizu H (2010). Plant responses to drought and rewatering. Plant Signal Behav.

[40] Ma Z, He S, Wang X, Sun J, Zhang Y, Zhang G (2018). Resequencing a core collection of upland cotton identifies genomic variation and loci influencing fiber quality and yield. Nat Genet.

[41] Wang P, Dong N, Wang M, Sun G, Jia Y, Geng X (2022). Introgression from Gossypium hirsutum is a driver for population divergence and genetic diversity in Gossypium barbadense. Plant J.

[42] Du X, Huang G, He S, Yang Z, Sun G, Ma X (2018). Resequencing of 243 diploid cotton accessions based on an updated a genome identifies the genetic basis of key agronomic traits. Nat Genet.

[43] Clarke JM, Townley-Smith TF (1986). Heritability and Relationship to yield of excised-leaf water retention in Durum wheat. Crop Sci.

[44] Bates LS, Waldren RP, Teare ID (1973). Rapid determination of free proline for water-stress studies. Plant Soil.

[45] Wijewardana C, Hock M, Henry B, Reddy KR (2015). Screening corn hybrids for cold tolerance using morphological traits for early-season seeding. Crop Sci.

