# Supplementary material for: Differential seedling growth and tolerance indices reflect drought tolerance in cotton
Source: BMC Plant Biol. 2022 Jul 11;22:331. doi: 10.1186/s12870-022-03724-4 (PMC9277823; doi:10.1186/s12870-022-03724-4)
Supplement: Supplementary file 1 — Additional file 1: Supplementary Figure S1. Biplot (Principal component analysis) of drought stress response indices (DSRI) for morpho-physiological and early root growth traits at the seedling stage. Scatter-plot shows the distribution of identified drought tolerant (purple) and susceptible (orang) cotton genotypes in (a). Correlation matrix heat map of shoot growth traits and root growth traits under NS (left) and DS (right) conditions in (b). Here PH, plant height; ChC, chlorophyll contents; LN, leaf number; LA, leaf area; LT leaf temperature; LFW, leaf fresh weight; LDW, leaf dry weight; LDW, leaf turgid weight; RWC, relative water contents; RL, root length; ARA, analyzed region area; ARW analyzed region width; ARH, analyzed region height; SA surface area; LPA, root length per area; AD average diameter; RV, root volume; RH, root heigh; RN root number; RF root forks; RC root crosses; RW root weight and R/S, root to shoot ratio. Supplementary Figure S2. Correlation among the drought stress response indices of growth rate (DSRI-GR) for 24 shoot and root growth traits in 6 cotton genotypes. Supplementary Figure S3. 18 cotton accessions sown in plastic cubes for shoot growth traits. Supplementary Figure S4. 18 cotton accessions sown in germination pouches for root growth traits. Supplementary Figure S5. Selected six cotton accessions sown in hydroponic condition for shoot and root growth traits. Supplementary Table S1. Details of accessions used in this study. Supplementary Table S2. Analysis of variance (ANOVA) of 18 cotton accession of three species including G. hirsutum, G. barbadense, and G. arboreum, for 18 shoot and root growth traits under control (NS) and PEG-induced drought stress (DS). Supplementary Table S3. Drought stress response index (DSRI) and cumulative drought stress response index (CDRI) of 18 cotton genotypes, including 6 genotypes from each three-cotton species Gossypium hirsutum, Gossypium barbadense, and Gossypium arboreum. Supplementary Table [file 12870_2022_3724_MOESM1_ESM.docx]

**Supplementary table S1.** Details of accessions used in this study

| Sr.# | ID | Code | Selected | New ID |
| --- | --- | --- | --- | --- |
| 1 | 182010-11 | Gh1 |  |  |
| 2 | 172384 | Gh2 | Susceptible | Gh-S |
| 3 | 172644 | Gh3 |  |  |
| 4 | 172662 | Gh4 |  |  |
| 5 | 172670 | Gh5 | Tolerant | Gh-T |
| 6 | 172665 | Gh6 |  |  |
| 7 | M310010 | Ga1 |  |  |
| 8 | M310087 | Ga2 |  |  |
| 9 | M310396 | Ga3 | Tolerant | Ga-T |
| 10 | M310183 | Ga4 |  |  |
| 11 | M310052 | Ga5 |  |  |
| 12 | M310080 | Ga6 | Susceptible | Ga-S |
| 13 | 178381 | Gb1 |  |  |
| 14 | 178245 | Gb2 |  |  |
| 15 | 178277 | Gb3 | Tolerant | Gb-T |
| 16 | 178293 | Gb4 | Susceptible | Gb-S |
| 17 | 178438 | Gb5 |  |  |
| 18 | 178419 | Gb6 |  |  |

**Supplementary Table S2.** Analysis of variance (ANOVA) of 18 cotton accession of three species including *G. hirsutum*, *G. barbadense,* and *G. arboreum,* for 18 shoot and root growth traits under control (NS) and PEG-induced drought stress (DS)

| Source | Rep | Geno | Trt | Geno*Trt | Error |
| --- | --- | --- | --- | --- | --- |
| DF | 2 | 17 | 1 | 17 | 70 |
| LT | 0.77 | 1.201** | 22.87** | 2.593** | 0.098 |
| LTW | 0.0009 | 0.044** | 0.183** | 0.002** | 0.0002 |
| LA | 23075 | 1745495** | 1.933** | 87546.7** | 10428.3 |
| LDW | 3.753 | 7.309** | 0.0025** | 8.567** | 9.692 |
| LFW | 0.0002 | 0.0382** | 0.3844** | 0.0027** | 0.0002 |
| LN | 0.049 | 1.769** | 21.33** | 0.710** | 0.073 |
| ChC | 5.71 | 72.95** | 750.5** | 8.56** | 1.53 |
| RWC | 1.1 | 64.6** | 13136** | 32.2** | 3 |
| PH | 0.426 | 209.6** | 615.1** | 5.425** | 0.303 |
| RL | 306 | 5407** | 116684** | 9092** | 227 |
| LPV | 706.7 | 5734.7** | 82691.0** | 8063.4** | 232.6 |
| AD | 1.37 | 139.4** | 94.90** | 23.79** | 1.18 |
| ARA | 192 | 78093** | 146347** | 40253** | 573 |
| PA | 752 | 101711** | 117854** | 24220** | 582 |
| RN | 109.3 | 1538** | 21646** | 2091** | 92.2 |
| RV | 7978 | 667141** | 1008 | 83631** | 4392 |
| RW | 0.0002 | 0.002** | 0.00006 | 0.003** | 0.0001 |
| SA | 749088 | 1136903** | 1381272** | 1169753** | 744404 |

NS = non-significant, * = Significant and ** = Significant at the 0.05

**Supplementary Table S3.** Drought stress response index (DSRI) and cumulative drought stress response index (CDRI) of 18 cotton genotypes, including 6 genotypes from each three-cotton species *Gossypium hirsutum*, *Gossypium barbadense,* and *Gossypium arboreum*.

|  | *Gh* | | | | | | *Gb* | | | | | | *Ga* | | | | | |
| --- | --- | --- | --- | --- | --- | --- | --- | --- | --- | --- | --- | --- | --- | --- | --- | --- | --- | --- |
|  | Gh1 | Gh2 | Gh3 | Gh4 | Gh5 | Gh6 | Gb1 | Gb2 | Gb3 | Gb4 | Gb5 | Gb6 | Ga1 | Ga2 | Ga3 | Ga4 | Ga5 | Ga6 |
| CDSRI-S | 26.55 | 22.74 | 26.89 | 26.65 | 30.77 | 25.49 | 25.79 | 28.23 | 30.32 | 21.24 | 24.44 | 24.18 | 23.46 | 21.86 | 26.10 | 21.01 | 20.23 | 18.21 |
| CDSRI-R | 9.72 | 7.42 | 10.81 | 11.64 | 13.72 | 8.73 | 8.26 | 11.36 | 12.27 | 6.84 | 8.95 | 7.63 | 7.17 | 6.21 | 10.00 | 5.21 | 3.91 | 2.61 |
| CDSRI | 18.14 | 15.08 | 18.85 | 19.14 | 22.24 | 17.11 | 17.02 | 19.80 | 21.30 | 14.04 | 16.70 | 15.90 | 15.31 | 14.03 | 18.05 | 13.11 | 12.07 | 10.41 |
| G% | 0.92 | 0.96 | 0.97 | 0.83 | 0.89 | 0.90 | 0.89 | 0.90 | 0.97 | 0.62 | 0.81 | 1.00 | 0.94 | 0.97 | 1.00 | 1.14 | 0.87 | 0.94 |
| PH | 0.63 | 0.82 | 0.72 | 0.75 | 0.69 | 0.63 | 0.86 | 0.79 | 0.80 | 0.75 | 0.63 | 0.73 | 0.72 | 0.66 | 0.71 | 0.72 | 0.69 | 0.65 |
| LA | 0.65 | 0.54 | 0.58 | 0.51 | 0.63 | 0.66 | 0.70 | 0.74 | 0.77 | 0.66 | 0.56 | 0.67 | 0.61 | 0.59 | 0.65 | 0.59 | 0.62 | 0.57 |
| LN | 1.14 | 0.72 | 0.67 | 0.86 | 0.69 | 1.09 | 0.70 | 0.67 | 0.96 | 0.68 | 0.69 | 0.65 | 0.93 | 0.80 | 0.73 | 0.63 | 0.93 | 0.89 |
| LFW | 0.63 | 0.48 | 0.55 | 0.47 | 0.67 | 0.57 | 0.67 | 0.68 | 0.72 | 0.55 | 0.57 | 0.64 | 0.55 | 0.55 | 0.60 | 0.53 | 0.57 | 0.51 |
| LTW | 0.79 | 0.60 | 0.71 | 0.59 | 0.84 | 0.76 | 0.94 | 0.81 | 0.89 | 0.66 | 0.74 | 0.79 | 0.66 | 0.66 | 0.72 | 0.62 | 0.69 | 0.62 |
| LDW | 0.80 | 0.64 | 0.91 | 0.58 | 0.97 | 0.78 | 1.01 | 0.92 | 1.00 | 0.64 | 0.75 | 0.91 | 0.73 | 0.62 | 0.71 | 0.60 | 0.79 | 0.64 |
| RWC | 0.70 | 0.76 | 0.68 | 0.77 | 0.75 | 0.76 | 0.75 | 0.81 | 0.79 | 0.67 | 0.72 | 0.77 | 0.79 | 0.79 | 0.79 | 0.84 | 0.78 | 0.76 |
| LT | 1.07 | 1.08 | 1.04 | 1.06 | 1.09 | 1.06 | 0.97 | 0.94 | 0.96 | 0.99 | 1.00 | 0.99 | 1.07 | 1.09 | 1.03 | 1.09 | 1.09 | 1.06 |
| ChC | 1.09 | 1.06 | 1.22 | 1.08 | 1.30 | 1.17 | 1.27 | 1.17 | 1.17 | 1.00 | 1.28 | 1.13 | 1.16 | 1.10 | 1.11 | 1.15 | 1.13 | 1.16 |
| RW | 1.31 | 1.88 | 1.06 | 1.22 | 1.24 | 2.14 | 1.96 | 3.59 | 1.86 | 1.79 | 0.38 | 1.80 | 0.34 | 0.37 | 0.22 | 0.33 | 0.54 | 0.45 |
| ARA | 0.66 | 0.72 | 1.29 | 1.36 | 1.49 | 0.64 | 0.45 | 0.99 | 1.44 | 0.29 | 1.33 | 0.62 | 0.80 | 1.08 | 1.23 | 0.68 | 0.45 | 0.18 |
| RL | 0.76 | 0.55 | 1.20 | 1.15 | 1.27 | 0.65 | 0.78 | 0.89 | 0.83 | 0.48 | 0.84 | 0.45 | 0.78 | 0.71 | 0.85 | 0.45 | 0.37 | 0.19 |
| PA | 0.63 | 0.59 | 1.42 | 1.45 | 1.62 | 0.76 | 1.01 | 1.04 | 1.38 | 0.60 | 0.85 | 0.73 | 0.94 | 0.45 | 1.24 | 0.43 | 0.46 | 0.20 |
| SA | 2.07 | 0.17 | 1.69 | 1.45 | 1.91 | 0.73 | 0.89 | 0.92 | 1.36 | 0.95 | 1.48 | 1.12 | 0.80 | 0.73 | 1.20 | 0.51 | 0.25 | 0.15 |
| AD | 0.91 | 1.39 | 1.07 | 1.15 | 1.24 | 1.13 | 0.91 | 1.05 | 1.72 | 0.95 | 1.08 | 0.89 | 1.18 | 0.90 | 1.51 | 1.25 | 1.06 | 0.89 |
| LPV | 0.75 | 0.53 | 1.40 | 1.21 | 1.33 | 0.75 | 1.01 | 0.85 | 0.75 | 0.54 | 0.97 | 0.65 | 0.73 | 0.59 | 0.90 | 0.46 | 0.27 | 0.23 |
| RV | 1.80 | 1.13 | 0.76 | 1.64 | 1.87 | 1.32 | 0.61 | 1.03 | 1.90 | 0.75 | 1.30 | 0.88 | 0.89 | 0.50 | 1.85 | 0.61 | 0.27 | 0.20 |
| RN | 0.82 | 0.43 | 0.92 | 1.02 | 1.74 | 0.61 | 0.65 | 1.01 | 1.04 | 0.49 | 0.73 | 0.47 | 0.71 | 0.87 | 1.01 | 0.48 | 0.24 | 0.13 |

**Supplementary Table S4.** Mean values of the shoot and root growth traits were measured at three different times, including zero days of stress (D0), after three days of stress (D3), and after six days of stress (D6) under NS DS conditions.

| Trt | Acc. | Time | | SL | RL | SD | LT | ChC | ChA | ChB | ChT | ChA/B | βC | PC | ARA | ARW | ARH | RL | LPA | SA | AD | S | RV | RH | RF | RC |
| --- | --- | --- | --- | --- | --- | --- | --- | --- | --- | --- | --- | --- | --- | --- | --- | --- | --- | --- | --- | --- | --- | --- | --- | --- | --- | --- |
| NS | Gh-T | | D0 | 17.3 | 17.5 | 2.3 | 27.1 | 24.1 | 22.0 | 15.1 | 29.5 | 1.3 | 0.1 | 0.3 | 14.2 | 3.8 | 5.1 | 56.1 | 13.5 | 42.5 | 2.2 | 64.1 | 2.4 | 397.7 | 1289.3 | 68.5 |
| NS | Gh-T | D3 | | 20.2 | 20.5 | 2.6 | 27.4 | 27.9 | 22.8 | 14.2 | 30.8 | 1.4 | 0.1 | 0.4 | 19.6 | 5.0 | 6.7 | 72.7 | 18.8 | 52.7 | 4.5 | 69.9 | 4.2 | 590.5 | 1386.5 | 73.0 |
| NS | Gh-T | D6 | | 22.3 | 22.4 | 2.7 | 26.6 | 30.5 | 24.7 | 19.9 | 37.3 | 1.2 | 0.1 | 0.4 | 25.0 | 5.4 | 7.7 | 74.1 | 21.1 | 58.8 | 5.0 | 83.4 | 5.0 | 743.5 | 1555.7 | 88.2 |
| NS | Gh-S | D0 | | 18.2 | 18.7 | 2.3 | 26.7 | 25.3 | 25.8 | 22.3 | 38.4 | 1.1 | 0.1 | 0.3 | 16.9 | 3.9 | 5.9 | 57.3 | 16.2 | 51.0 | 2.8 | 59.3 | 3.6 | 424.0 | 1290.8 | 57.8 |
| NS | Gh-S | D3 | | 20.7 | 23.5 | 2.6 | 27.3 | 28.5 | 26.8 | 23.3 | 40.4 | 1.1 | 0.1 | 0.3 | 21.9 | 5.1 | 7.2 | 76.8 | 21.0 | 59.5 | 3.5 | 76.8 | 4.5 | 528.8 | 1383.7 | 64.7 |
| NS | Gh-S | D6 | | 22.7 | 26.1 | 2.9 | 27.0 | 30.6 | 25.6 | 22.9 | 40.6 | 1.1 | 0.1 | 0.3 | 22.9 | 5.3 | 7.5 | 86.1 | 20.8 | 56.6 | 4.2 | 84.6 | 4.4 | 692.5 | 1475.2 | 66.3 |
| NS | Gb-T | D0 | | 16.8 | 16.1 | 2.3 | 25.5 | 25.9 | 22.3 | 15.3 | 29.8 | 1.4 | 0.2 | 0.2 | 12.2 | 3.7 | 4.5 | 56.5 | 11.6 | 36.3 | 1.9 | 55.0 | 1.9 | 499.3 | 991.2 | 56.3 |
| NS | Gb-T | D3 | | 20.6 | 18.7 | 2.6 | 27.0 | 28.6 | 23.8 | 17.0 | 34.8 | 1.3 | 0.2 | 0.3 | 17.3 | 4.7 | 6.4 | 70.7 | 16.6 | 45.6 | 4.2 | 68.7 | 3.4 | 582.2 | 1220.0 | 70.8 |
| NS | Gb-T | D6 | | 22.1 | 21.4 | 2.8 | 26.4 | 29.9 | 25.6 | 20.7 | 38.5 | 1.2 | 0.2 | 0.3 | 24.2 | 5.2 | 7.2 | 71.7 | 17.9 | 48.7 | 5.3 | 89.2 | 3.6 | 685.0 | 1471.5 | 91.0 |
| NS | Gb-S | D0 | | 20.7 | 16.5 | 2.4 | 26.0 | 24.5 | 24.3 | 18.3 | 33.9 | 1.3 | 0.2 | 0.2 | 12.2 | 3.4 | 5.0 | 64.5 | 11.5 | 36.2 | 1.8 | 65.5 | 1.6 | 489.2 | 1180.0 | 90.8 |
| NS | Gb-S | D3 | | 23.7 | 21.2 | 2.7 | 26.9 | 26.4 | 24.3 | 18.6 | 35.4 | 1.3 | 0.2 | 0.3 | 20.0 | 4.9 | 6.6 | 90.1 | 18.6 | 52.1 | 3.8 | 91.1 | 3.3 | 601.5 | 1801.2 | 120.3 |
| NS | Gb-S | D6 | | 24.5 | 24.3 | 3.1 | 27.1 | 28.5 | 27.3 | 24.0 | 41.6 | 1.2 | 0.1 | 0.3 | 24.1 | 5.2 | 7.3 | 96.8 | 19.2 | 52.9 | 4.1 | 93.8 | 3.7 | 758.3 | 1871.7 | 137.3 |
| NS | Ga-T | D0 | | 12.3 | 19.3 | 1.6 | 25.9 | 21.7 | 26.0 | 25.0 | 38.9 | 1.0 | 0.1 | 0.3 | 10.7 | 3.7 | 4.0 | 39.6 | 10.4 | 32.6 | 2.3 | 39.6 | 2.0 | 162.7 | 720.0 | 39.8 |
| NS | Ga-T | D3 | | 14.8 | 20.4 | 1.9 | 27.2 | 26.7 | 26.7 | 24.2 | 40.1 | 1.1 | 0.1 | 0.3 | 19.6 | 4.1 | 7.4 | 46.5 | 18.3 | 51.0 | 4.5 | 44.0 | 3.8 | 240.0 | 741.3 | 33.2 |
| NS | Ga-T | D6 | | 16.2 | 22.7 | 2.1 | 26.2 | 28.9 | 26.9 | 23.7 | 41.0 | 1.1 | 0.2 | 0.3 | 23.9 | 5.2 | 7.5 | 59.4 | 18.4 | 50.2 | 5.3 | 48.9 | 4.8 | 545.5 | 868.3 | 44.5 |
| NS | Ga-S | D0 | | 12.3 | 13.8 | 2.0 | 26.8 | 24.4 | 26.0 | 25.4 | 39.2 | 1.0 | 0.1 | 0.2 | 9.3 | 3.2 | 4.0 | 38.1 | 9.0 | 28.2 | 2.4 | 38.1 | 1.7 | 144.5 | 773.5 | 40.2 |
| NS | Ga-S | D3 | | 14.8 | 17.3 | 2.2 | 27.4 | 29.8 | 27.2 | 28.1 | 43.9 | 1.0 | 0.0 | 0.4 | 17.5 | 4.4 | 6.2 | 48.3 | 16.0 | 43.9 | 3.4 | 48.3 | 3.6 | 231.5 | 967.8 | 53.2 |
| NS | Ga-S | D6 | | 16.7 | 19.4 | 2.4 | 26.3 | 34.3 | 27.7 | 27.3 | 44.8 | 1.0 | 0.1 | 0.2 | 20.9 | 5.0 | 6.6 | 53.1 | 16.9 | 45.6 | 4.2 | 55.1 | 4.1 | 361.8 | 1035.8 | 52.8 |
| DS | Gh-T | D0 | | 17.5 | 18.8 | 2.1 | 26.5 | 24.7 | 21.2 | 14.1 | 28.1 | 1.4 | 0.1 | 0.3 | 15.1 | 3.6 | 5.6 | 58.8 | 14.4 | 45.3 | 2.6 | 58.8 | 3.0 | 399.2 | 1224.8 | 53.7 |
| DS | Gh-T | D3 | | 17.6 | 23.3 | 2.5 | 27.5 | 30.0 | 27.6 | 21.0 | 39.2 | 1.2 | 0.1 | 1.0 | 20.0 | 5.0 | 7.3 | 69.4 | 19.7 | 53.0 | 3.5 | 66.4 | 4.1 | 454.3 | 1164.9 | 54.4 |
| DS | Gh-T | D6 | | 19.2 | 25.2 | 2.6 | 28.4 | 33.1 | 29.0 | 27.6 | 48.6 | 1.0 | 0.1 | 1.9 | 24.9 | 5.7 | 7.9 | 78.6 | 24.2 | 65.3 | 6.8 | 63.6 | 9.8 | 618.0 | 1257.3 | 57.5 |
| DS | Gh-S | D0 | | 17.6 | 15.1 | 2.2 | 25.1 | 25.8 | 25.8 | 22.3 | 38.4 | 1.1 | 0.1 | 0.3 | 12.9 | 3.6 | 4.8 | 62.5 | 11.7 | 40.1 | 5.7 | 62.5 | 2.9 | 411.5 | 1216.3 | 52.0 |
| DS | Gh-S | D3 | | 18.0 | 17.0 | 2.5 | 27.6 | 30.6 | 27.8 | 24.3 | 38.7 | 1.1 | 0.1 | 0.6 | 16.4 | 4.3 | 6.0 | 65.4 | 15.7 | 42.8 | 2.9 | 69.4 | 3.0 | 447.5 | 1179.3 | 79.8 |
| DS | Gh-S | D6 | | 19.6 | 17.9 | 2.6 | 28.6 | 31.8 | 28.7 | 32.3 | 44.4 | 0.9 | 0.0 | 0.7 | 17.4 | 4.8 | 6.7 | 53.8 | 16.3 | 42.0 | 3.5 | 65.2 | 3.6 | 481.8 | 1067.3 | 66.5 |
| DS | Gb-T | D0 | | 17.8 | 20.3 | 2.3 | 23.9 | 26.3 | 22.6 | 15.6 | 30.3 | 1.3 | 0.2 | 0.3 | 12.5 | 3.7 | 5.0 | 61.0 | 12.8 | 41.2 | 1.8 | 70.5 | 1.7 | 502.2 | 1092.6 | 71.7 |
| DS | Gb-T | D3 | | 18.9 | 24.5 | 2.6 | 27.2 | 30.5 | 26.8 | 19.6 | 38.9 | 1.3 | 0.2 | 0.6 | 20.9 | 4.6 | 7.5 | 68.9 | 20.2 | 56.4 | 3.7 | 69.9 | 5.0 | 622.7 | 1372.7 | 83.7 |
| DS | Gb-T | D6 | | 21.2 | 26.8 | 2.7 | 27.3 | 33.5 | 28.1 | 23.4 | 42.3 | 1.2 | 0.2 | 1.0 | 24.0 | 5.6 | 8.0 | 79.4 | 23.2 | 62.0 | 4.3 | 74.4 | 5.5 | 583.7 | 1281.2 | 76.2 |
| DS | Gb-S | D0 | | 20.4 | 14.3 | 2.4 | 25.3 | 24.4 | 23.8 | 17.3 | 32.6 | 1.3 | 0.2 | 0.3 | 11.3 | 3.8 | 4.3 | 51.5 | 11.3 | 35.5 | 2.3 | 51.5 | 2.0 | 275.2 | 1138.8 | 67.7 |
| DS | Gb-S | D3 | | 21.0 | 16.7 | 2.6 | 27.9 | 27.8 | 27.3 | 22.4 | 40.4 | 1.2 | 0.2 | 0.6 | 14.7 | 4.5 | 5.6 | 60.5 | 14.1 | 37.8 | 2.9 | 62.0 | 2.7 | 421.7 | 1007.0 | 70.2 |
| DS | Gb-S | D6 | | 21.3 | 16.9 | 2.7 | 28.3 | 31.0 | 25.3 | 22.6 | 40.3 | 1.0 | 0.2 | 0.6 | 15.9 | 4.7 | 6.2 | 63.9 | 15.2 | 37.0 | 3.3 | 61.9 | 3.4 | 459.5 | 833.0 | 54.5 |
| DS | Ga-T | D0 | | 13.2 | 21.0 | 1.5 | 25.6 | 26.2 | 25.9 | 24.1 | 40.0 | 1.0 | 0.1 | 0.3 | 13.5 | 3.3 | 5.9 | 48.3 | 13.2 | 41.3 | 2.8 | 48.3 | 3.1 | 183.5 | 826.2 | 45.0 |
| DS | Ga-T | D3 | | 13.7 | 22.9 | 1.8 | 27.5 | 30.4 | 29.2 | 29.8 | 44.0 | 1.0 | 0.1 | 0.6 | 18.4 | 4.6 | 6.9 | 57.1 | 18.9 | 48.9 | 4.4 | 52.1 | 5.0 | 311.7 | 664.5 | 33.0 |
| DS | Ga-T | D6 | | 14.3 | 27.0 | 2.0 | 27.9 | 32.4 | 28.8 | 27.1 | 44.0 | 1.1 | 0.1 | 0.9 | 22.2 | 5.2 | 7.8 | 62.8 | 21.2 | 54.6 | 4.7 | 53.3 | 5.7 | 363.8 | 702.3 | 37.0 |
| DS | Ga-S | D0 | | 12.1 | 16.6 | 1.7 | 27.3 | 30.7 | 26.1 | 26.2 | 40.0 | 1.0 | 0.0 | 0.3 | 9.9 | 3.3 | 4.6 | 33.0 | 9.6 | 31.9 | 2.9 | 34.0 | 2.3 | 131.0 | 807.0 | 42.7 |
| DS | Ga-S | D3 | | 13.4 | 18.4 | 2.1 | 27.6 | 33.9 | 27.8 | 25.7 | 45.3 | 1.1 | 0.1 | 0.6 | 15.8 | 4.3 | 6.5 | 41.6 | 15.3 | 41.5 | 3.3 | 52.1 | 3.2 | 267.8 | 875.2 | 54.2 |
| DS | Ga-S | D6 | | 13.6 | 19.0 | 2.3 | 27.7 | 36.2 | 28.1 | 29.7 | 45.4 | 0.9 | 0.0 | 0.7 | 16.3 | 4.4 | 7.1 | 44.8 | 16.3 | 40.4 | 4.0 | 51.8 | 3.8 | 264.7 | 952.8 | 60.5 |

**Supplementary Table S5.** Growth rate (GR) of indivisual shoot and root growth traits were measured at three different times, including zero days of stress (D0), after three days of stress (D3), and after six days of stress (D6) under NS DS conditions.

| Trt. | Stage | Gen. | CRGR | SL | SD | RL | RL | ARA | ARW | ARH | PA | SA | AD | RLV | RV | RN | RF | RC | LT | ChC | ChA | ChB | ChT | ChA/B | β– C | PC |
| --- | --- | --- | --- | --- | --- | --- | --- | --- | --- | --- | --- | --- | --- | --- | --- | --- | --- | --- | --- | --- | --- | --- | --- | --- | --- | --- |
| NS | D3 | Gh-T | 5.60 | 0.16 | 0.13 | 0.17 | 0.30 | 0.39 | 0.29 | 0.30 | 0.39 | 0.24 | 1.03 | 0.09 | 0.72 | 0.48 | 0.08 | 0.07 | 0.01 | 0.16 | 0.03 | -0.06 | 0.04 | 0.04 | 0.23 | 0.32 |
| NS | D3 | Gh-S | 4.09 | 0.14 | 0.11 | 0.26 | 0.34 | 0.30 | 0.32 | 0.22 | 0.29 | 0.17 | 0.24 | 0.30 | 0.25 | 0.25 | 0.07 | 0.12 | 0.03 | 0.13 | 0.04 | 0.05 | 0.05 | 0.00 | 0.09 | 0.34 |
| NS | D3 | Gb-T | 6.08 | 0.22 | 0.14 | 0.16 | 0.25 | 0.42 | 0.28 | 0.44 | 0.43 | 0.26 | 1.18 | 0.25 | 0.84 | 0.17 | 0.23 | 0.13 | 0.03 | 0.11 | 0.07 | 0.11 | 0.17 | -0.02 | -0.01 | 0.22 |
| NS | D3 | Gb-S | 7.51 | 0.15 | 0.12 | 0.28 | 0.40 | 0.64 | 0.45 | 0.34 | 0.62 | 0.44 | 1.16 | 0.39 | 1.07 | 0.23 | 0.43 | 0.13 | 0.03 | 0.08 | 0.00 | 0.02 | 0.04 | 0.04 | 0.15 | 0.31 |
| NS | D3 | Ga-T | 6.27 | 0.20 | 0.16 | 0.06 | 0.18 | 0.73 | 0.11 | 0.56 | 0.57 | 0.46 | 0.95 | 0.11 | 0.90 | 0.48 | 0.03 | -0.17 | 0.05 | 0.23 | 0.03 | -0.03 | 0.03 | 0.06 | 0.29 | 0.29 |
| NS | D3 | Ga-S | 7.44 | 0.21 | 0.09 | 0.26 | 0.27 | 0.78 | 0.38 | 0.55 | 0.59 | 0.46 | 0.45 | 0.27 | 1.13 | 0.60 | 0.25 | 0.32 | 0.02 | 0.22 | 0.05 | 0.11 | 0.12 | -0.05 | -0.40 | 0.77 |
| NS | D6 | Gh-T | 9.21 | 0.28 | 0.18 | 0.28 | 0.32 | 0.77 | 0.41 | 0.50 | 0.56 | 0.39 | 1.24 | 0.30 | 1.07 | 0.87 | 0.21 | 0.29 | 0.02 | 0.26 | 0.12 | 0.32 | 0.26 | -0.09 | 0.38 | 0.26 |
| NS | D6 | Gh-S | 6.71 | 0.25 | 0.23 | 0.40 | 0.50 | 0.55 | 0.38 | 0.37 | 0.48 | 0.21 | 0.49 | 0.43 | 0.82 | 0.63 | 0.14 | 0.15 | 0.01 | 0.21 | 0.05 | 0.03 | 0.06 | 0.01 | 0.08 | 0.24 |
| NS | D6 | Gb-T | 9.86 | 0.31 | 0.23 | 0.33 | 0.27 | 0.98 | 0.40 | 0.62 | 0.55 | 0.34 | 1.75 | 0.62 | 0.92 | 0.37 | 0.48 | 0.62 | 0.03 | 0.16 | 0.15 | 0.35 | 0.29 | -0.09 | -0.11 | 0.28 |
| NS | D6 | Gb-S | 10.06 | 0.19 | 0.28 | 0.47 | 0.50 | 0.97 | 0.53 | 0.47 | 0.67 | 0.46 | 1.32 | 0.43 | 1.30 | 0.55 | 0.59 | 0.51 | 0.04 | 0.16 | 0.12 | 0.31 | 0.23 | -0.07 | -0.24 | 0.26 |
| NS | D6 | Ga-T | 9.43 | 0.32 | 0.27 | 0.17 | 0.50 | 0.83 | 0.39 | 0.88 | 0.78 | 0.54 | 1.32 | 0.23 | 1.01 | 0.84 | 0.21 | 0.12 | 0.01 | 0.33 | 0.03 | -0.05 | 0.06 | 0.13 | 0.24 | 0.28 |
| NS | D6 | Ga-S | 9.63 | 0.36 | 0.20 | 0.41 | 0.40 | 0.88 | 0.57 | 0.66 | 0.89 | 0.62 | 0.77 | 0.45 | 1.06 | 0.81 | 0.34 | 0.32 | 0.02 | 0.40 | 0.07 | 0.08 | 0.14 | 0.02 | 0.09 | 0.08 |
| DS | D3 | Gh-T | 6.29 | 0.00 | 0.17 | 0.24 | 0.18 | 0.32 | 0.37 | 0.30 | 0.37 | 0.17 | 0.36 | 0.13 | 0.37 | 0.14 | -0.05 | 0.01 | 0.04 | 0.21 | 0.08 | 0.49 | 0.39 | -0.10 | 0.09 | 2.00 |
| DS | D3 | Gh-S | 2.42 | 0.02 | 0.12 | 0.13 | 0.05 | 0.28 | 0.19 | 0.24 | 0.34 | 0.07 | -0.49 | 0.11 | 0.04 | 0.09 | -0.03 | 0.04 | 0.10 | 0.19 | 0.08 | 0.09 | 0.01 | -0.01 | -0.13 | 0.92 |
| DS | D3 | Gb-T | 7.35 | 0.06 | 0.13 | 0.20 | 0.13 | 0.67 | 0.24 | 0.48 | 0.58 | 0.37 | 1.01 | -0.01 | 0.96 | 0.24 | 0.06 | 0.17 | 0.04 | 0.16 | 0.19 | 0.25 | 0.28 | -0.05 | 0.01 | 1.19 |
| DS | D3 | Gb-S | 4.67 | 0.03 | 0.10 | 0.16 | 0.17 | 0.30 | 0.21 | 0.31 | 0.24 | 0.06 | 0.28 | 0.10 | 0.35 | 0.53 | -0.12 | 0.04 | 0.10 | 0.14 | 0.15 | 0.29 | 0.24 | -0.09 | -0.21 | 1.28 |
| DS | D3 | Ga-T | 4.99 | 0.04 | 0.20 | 0.09 | 0.18 | 0.36 | 0.37 | 0.17 | 0.43 | 0.18 | 0.57 | 0.08 | 0.60 | 0.70 | -0.20 | -0.27 | 0.08 | 0.16 | 0.13 | 0.24 | 0.10 | -0.07 | -0.38 | 1.24 |
| DS | D3 | Ga-S | 6.70 | 0.11 | 0.27 | 0.11 | 0.26 | 0.60 | 0.29 | 0.41 | 0.59 | 0.30 | 0.15 | 0.15 | 0.39 | 0.80 | 0.08 | 0.27 | 0.01 | 0.10 | 0.07 | -0.02 | 0.13 | 0.09 | 0.75 | 0.79 |
| DS | D6 | Gh-T | 15.76 | 0.10 | 0.25 | 0.34 | 0.34 | 0.65 | 0.57 | 0.41 | 0.68 | 0.44 | 1.62 | 0.08 | 2.27 | 0.55 | 0.03 | 0.07 | 0.07 | 0.34 | 0.37 | 0.95 | 0.73 | -0.25 | 0.31 | 4.86 |
| DS | D6 | Gh-S | 4.71 | 0.11 | 0.17 | 0.19 | 0.14 | 0.35 | 0.34 | 0.40 | 0.39 | 0.05 | 0.40 | 0.04 | 0.24 | 0.17 | 0.01 | 0.28 | 0.14 | 0.23 | 0.11 | 0.45 | 0.16 | -0.20 | -0.80 | 1.35 |
| DS | D6 | Gb-T | 12.50 | 0.19 | 0.17 | 0.32 | 0.30 | 0.91 | 0.52 | 0.59 | 0.81 | 0.51 | 1.32 | 0.06 | 2.23 | 0.16 | 0.17 | 0.06 | 0.14 | 0.28 | 0.25 | 0.50 | 0.39 | -0.14 | -0.13 | 2.91 |
| DS | D6 | Gb-S | 6.18 | 0.04 | 0.14 | 0.18 | 0.24 | 0.41 | 0.26 | 0.46 | 0.34 | 0.04 | 0.46 | 0.20 | 0.73 | 0.67 | 0.03 | 0.02 | 0.12 | 0.27 | 0.06 | 0.31 | 0.23 | -0.21 | -0.10 | 1.27 |
| DS | D6 | Ga-T | 9.37 | 0.09 | 0.31 | 0.29 | 0.30 | 0.64 | 0.55 | 0.31 | 0.61 | 0.32 | 0.98 | 0.10 | 0.83 | 0.98 | 0.15 | 0.18 | 0.09 | 0.23 | 0.11 | 0.13 | 0.10 | 0.01 | -0.15 | 2.21 |
| DS | D6 | Ga-S | 6.64 | 0.13 | 0.18 | 0.15 | 0.36 | 0.65 | 0.33 | 0.54 | 0.49 | 0.27 | 0.38 | 0.22 | 0.62 | 0.96 | 0.11 | 0.22 | 0.01 | 0.18 | 0.08 | 0.13 | 0.13 | -0.04 | -0.67 | 1.22 |


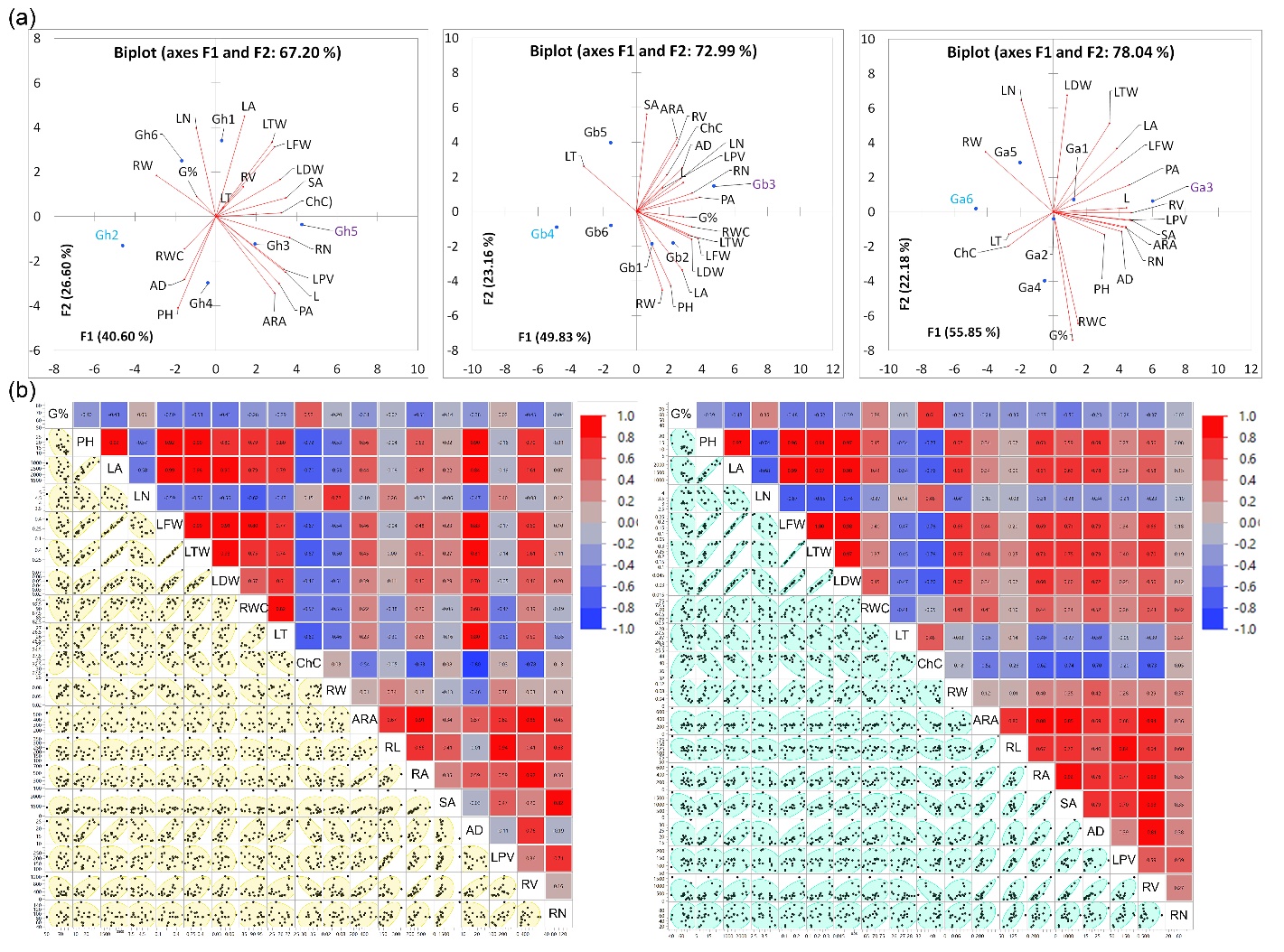


**Supplementary Figure S1.** Biplot (Principal component analysis) of drought stress response indices (DSRI) for morpho-physiological and early root growth traits at the seedling stage. Scatter-plot shows the distribution of identified drought tolerant (purple) and susceptible (orang) cotton genotypes in (a). Correlation matrix heat map of shoot growth traits and root growth traits under NS (left) and DS (right) conditions in (b). Here PH, plant height; ChC, chlorophyll contents; LN, leaf number; LA, leaf area; LT leaf temperature; LFW, leaf fresh weight; LDW, leaf dry weight; LDW, leaf turgid weight; RWC, relative water contents; RL, root length; ARA, analyzed region area; ARW analyzed region width; ARH, analyzed region height; SA surface area; LPA, root length per area; AD average diameter; RV, root volume; RH, root heigh; RN root number; RF root forks; RC root crosses; RW root weight and R/S, root to shoot ratio.


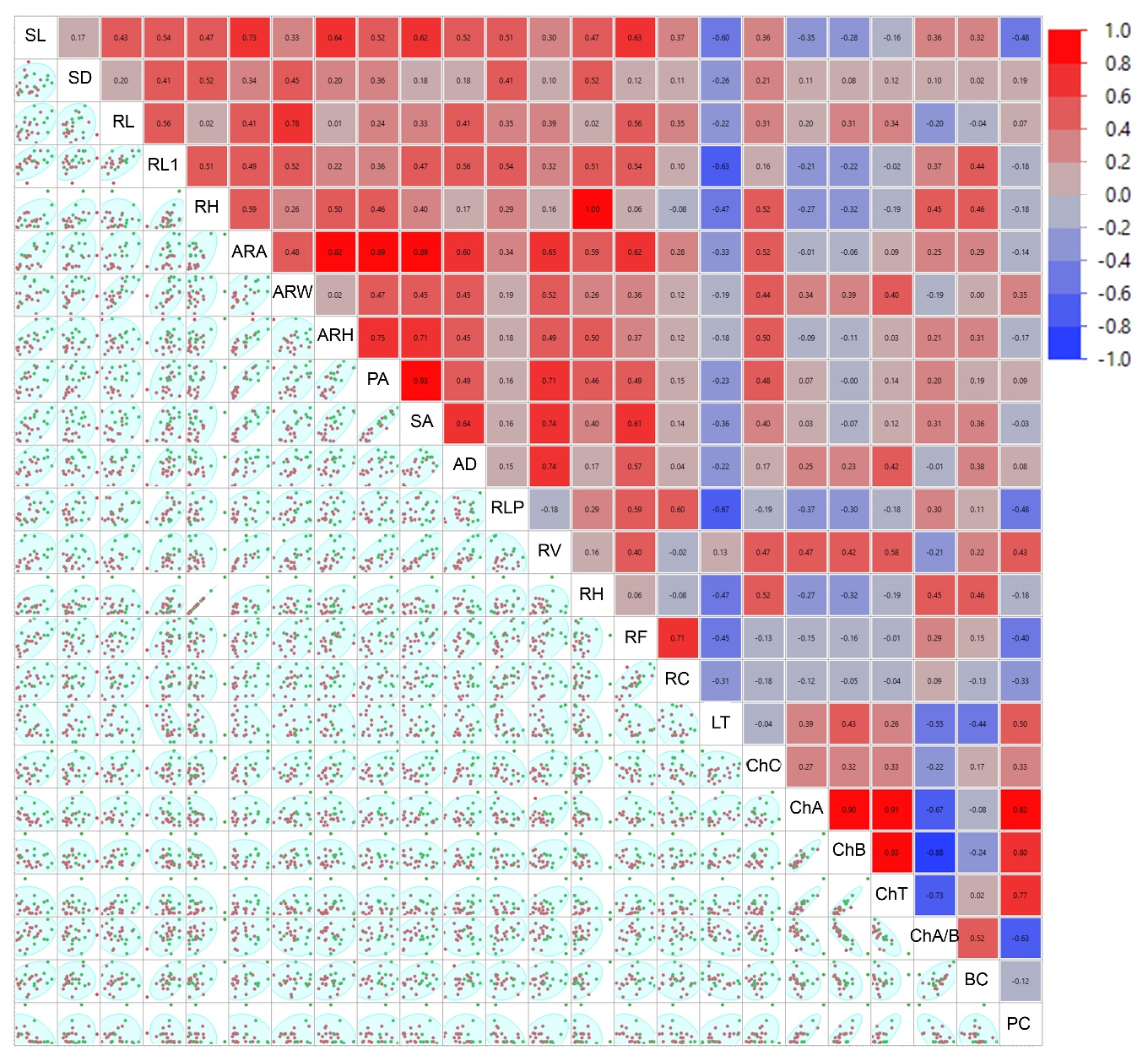


**Supplementary Figure S2.** Correlation among the drought stress response indices of growth rate (DSRI-GR) for 24 shoot and root growth traits in 6 cotton genotypes.

**
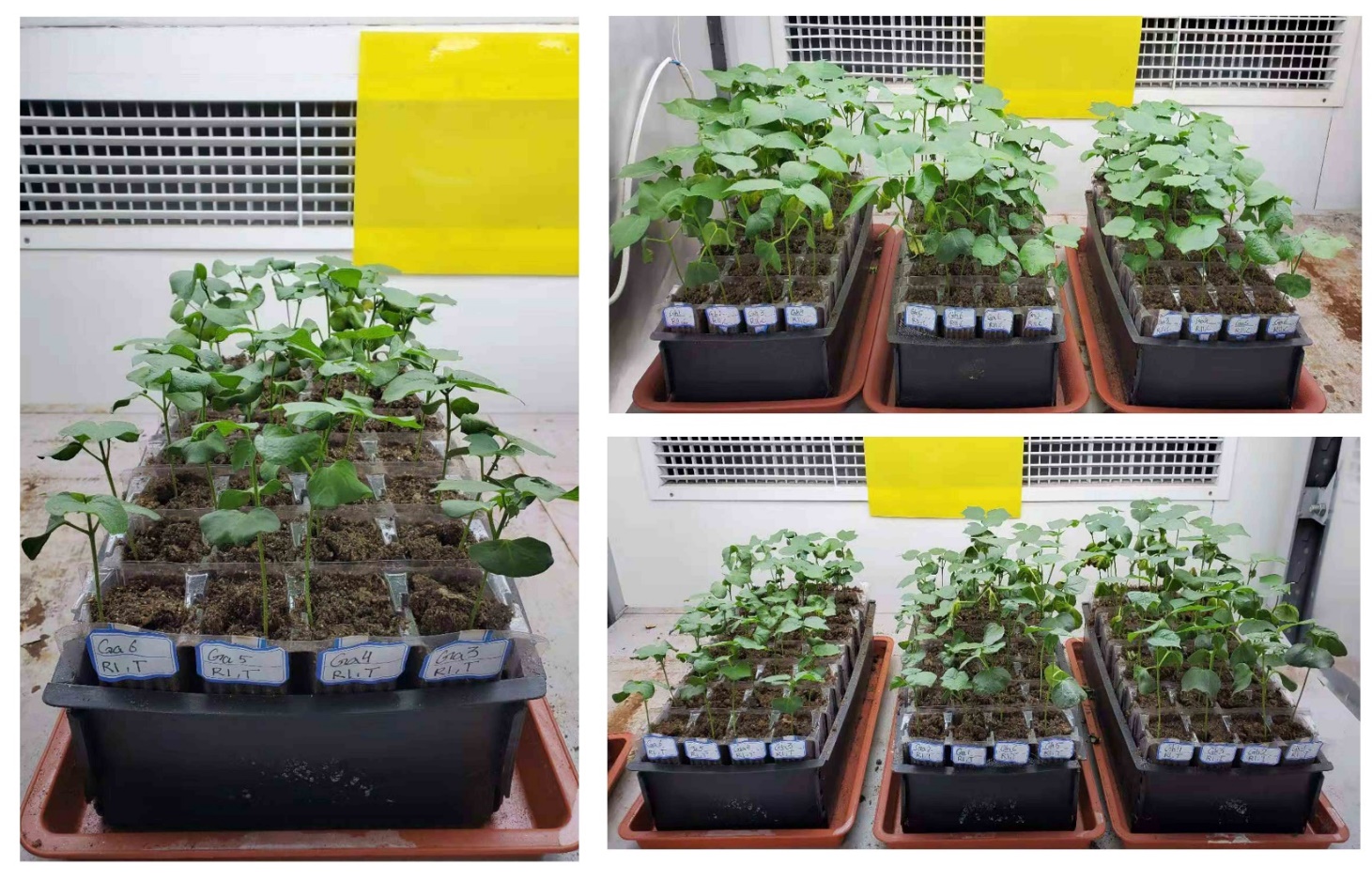
**

**Supplementary Figure S3.** 18 cotton accessions sown in plastic cubes for shoot growth traits

**
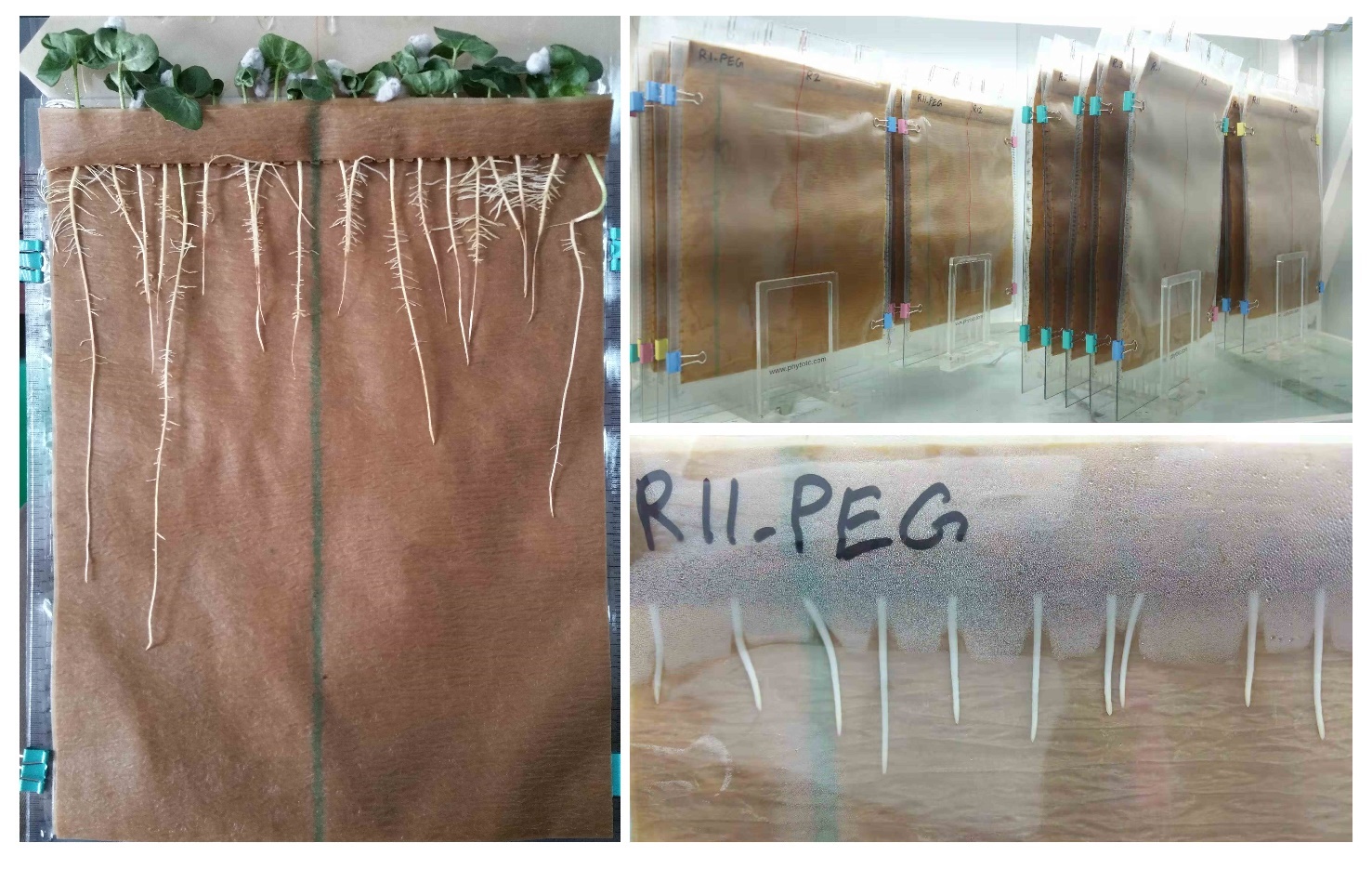
**

**Supplementary Figure S4.** 18 cotton accessions sown in germination pouches for root growth traits

**
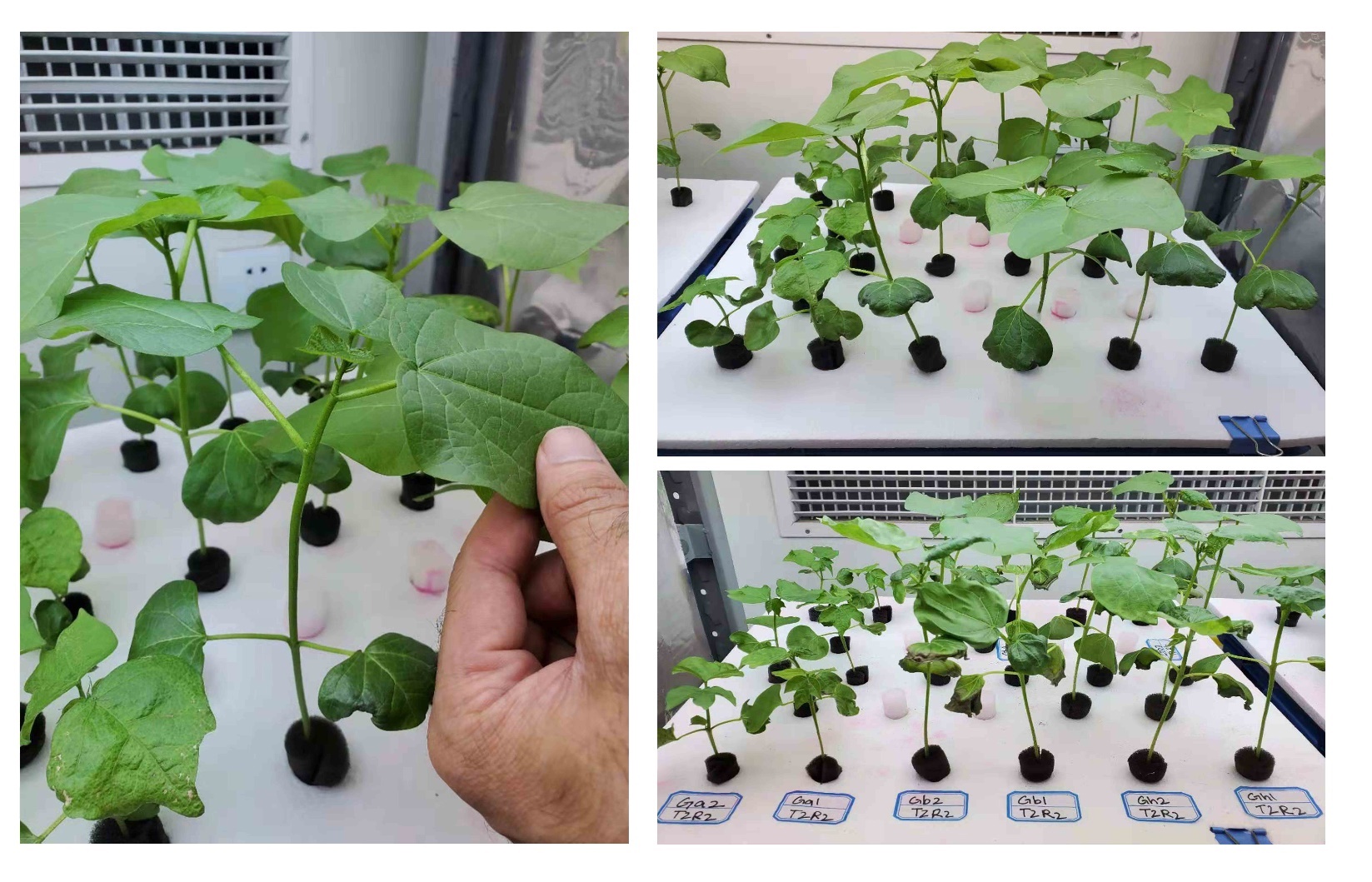
**

**Supplementary Figure S5.** Selected six cotton accessions sown in hydroponic condition for shoot and root growth traits
